# The Effects of Nano-Curcumin Supplementation on Risk Factors for Cardiovascular Disease: A GRADE-Assessed Systematic Review and Meta-Analysis of Clinical Trials

**DOI:** 10.3390/antiox10071015

**Published:** 2021-06-24

**Authors:** Damoon Ashtary-Larky, Mahnaz Rezaei Kelishadi, Reza Bagheri, Seyedeh Parisa Moosavian, Alexei Wong, Sayed Hossein Davoodi, Pardis Khalili, Frédéric Dutheil, Katsuhiko Suzuki, Omid Asbaghi

**Affiliations:** 1 Nutrition and Metabolic Diseases Research Center, Ahvaz Jundishapur University of Medical Sciences, Ahvaz 6135715794, Iran; damoon_ashtary@yahoo.com; 2 Department of Community Nutrition, School of Nutrition and Food Science, Isfahan University of Medical Sciences, Isfahan 8174673461, Iran; m.rezaei81@yahoo.com; 3 Department of Exercise Physiology, University of Isfahan, Isfahan 8174673441, Iran; will.fivb@yahoo.com; 4 Department of Clinical Nutrition, School of Nutrition and Food Science, Isfahan University of Medical Sciences, Isfahan 8174673461, Iran; p_moosavian@yahoo.com; 5 Department of Health and Human Performance, Marymount University, Arlington, VA 22207, USA; awong@marymount.edu; 6 Cancer Research Center, Shahid Beheshti University of Medical Sciences, Tehran 1416753955, Iran; reza.bagheri@alumni.um.ac.ir; 7 Department of Clinical Nutrition and Dietetics, Faculty of Nutrition Sciences and Food Technology, National Nutrition and Food Technology, Research Institute, Shahid Beheshti University of Medical Sciences, Tehran 1416753955, Iran; khalilipardis73@gmail.com; 8 CNRS, LaPSCo, Physiological and Psychosocial Stress, CHU Clermont-Ferrand, University Hospital of Clermont-Ferrand, Preventive and Occupational Medicine, WittyFit, Université Clermont Auvergne, F-63000 Clermont-Ferrand, France; fred_dutheil@yahoo.fr; 9 Faculty of Sport Sciences, Waseda University, 2-579-15 Mikajima, Tokorozawa 359-1192, Japan

**Keywords:** nano-curcumin, curcumin, cardiovascular risk, meta-analysis, systematic review

## Abstract

Previous studies have indicated that curcumin supplementation may be beneficial for cardiometabolic health; however, current evidence regarding the effects of its nanorange formulations, popularly known as “nano-curcumin”, remains unclear. This systematic review and meta-analysis aimed to determine the impact of nano-curcumin supplementation on risk factors for cardiovascular disease. PubMed, Scopus, Embase, and ISI web of science were systematically searched up to May 2021 using relevant keywords. All randomized controlled trials (RCTs) investigating the effects of nano-curcumin supplementation on cardiovascular disease risk factors were included. Meta-analysis was performed using random-effects models, and subgroup analysis was performed to explore variations by dose and baseline risk profiles. According to the results of this study, nano-curcumin supplementation was associated with improvements in the glycemic profile by decreasing fasting blood glucose (FBG) (WMD: −18.14 mg/dL; 95% CI: −29.31 to −6.97; *p* = 0.001), insulin (WMD: −1.21 mg/dL; 95% CI: −1.43 to −1.00; *p* < 0.001), and HOMA-IR (WMD: −0.28 mg/dL; 95% CI: −0.33 to −0.23; *p* < 0.001). Interestingly, nano-curcumin supplementation resulted in increases in high-density lipoprotein (HDL) (WMD: 5.77 mg/dL; 95% CI: 2.90 to 8.64; *p* < 0.001). In terms of other lipid profile markers (triglyceride (TG), total cholesterol (TC), and low-density lipoprotein (LDL)), subgroup analyses showed that nano-curcumin supplementation had more favorable effects on lipid profiles in individuals with dyslipidemia at baseline. Nano-curcumin supplementation also showed favorable anti-inflammatory effects by decreasing C-reactive protein (CRP) (WMD: −1.29 mg/L; 95% CI: −2.15 to −0.44; *p* = 0.003) and interleukin-6 (IL-6) (WMD: −2.78 mg/dL; 95% CI: −3.76 to −1.79; *p* < 0.001). Moreover, our results showed the hypotensive effect of nano-curcumin, evidenced by a decrease in systolic blood pressure (SBP). In conclusion, our meta-analysis suggests that nano-curcumin supplementation may decline cardiovascular disease risk by improving glycemic and lipid profiles, inflammation, and SBP. Future large-scale investigations with longer durations are needed to expand on our findings.

## 1. Introduction

Cardiovascular disease (CVD) is the leading cause of death worldwide, placing heavy economic and health burdens on society [1]. Risk factors for CVD such as obesity, hypertension, diabetes, metabolic syndrome, nonalcoholic fatty liver disease, and dyslipidemia lead to increased atherosclerosis [2,3], considered by many to be the most important component in CVD pathologies. Consequently, improving the risk factors for CVD disease is essential to lessen the morbidity and mortality of this condition. Although it is well known that a wide range of pharmacotherapies may improve CVD and its risk factors, these are shown to produce side effects and complications in some individuals. Therefore, nutraceutical therapies such as dietary supplements could be considered alternative or adjunct treatments for CVD [4,5].

Curcumin is a medicinal plant often used as a dietary supplement, which has been shown to improve cardiovascular function [6,7]. Curcumin is the main natural polyphenol found in the rhizome of Curcuma longa (turmeric) and other Curcuma species [8]. During the last five decades, it has been revealed that most of the effects of Curcuma longa are mainly due to curcumin, with potential effects against various metabolic and inflammatory diseases such as diabetes, allergies, arthritis, Alzheimer’s disease, and other chronic illnesses [9]. Moreover, the therapeutic effects of curcumin have been investigated in the treatment of CVD [10]. It seems that curcumin exerts its cardiovascular protective effects by its anti-inflammatory, antioxidant, antiproliferative, antilipidemic, and antithrombotic properties [11].

Although curcumin has been shown to have positive effects on CVD and its risk factors, it exhibits poor solubility in water, and therefore the systemic bioavailability is also very low. This is attributed to very poor absorption, faster metabolism, and systemic elimination after oral administration [12]. Consequently, its therapeutic actions are also significantly diminished. Therefore, the nanorange formulations of curcumin, popularly known as “nano-curcumin” have been designed to overcome this limitation [13,14]. Although both curcumin and nano-curcumin have the same chemical structure, theoretically, nano-curcumin may be effective, if not more effective, than curcumin at improving the risk factors for CVD. For example, some previous in vitro and in vivo studies revealed that nano-curcumin might be superior to native curcumin in some therapeutic properties [15,16]. Basniwal et al. demonstrated that under aqueous conditions, nano-curcumin could exhibit a similar or a much stronger antiproliferative effect on cancer cells compared to normal curcumin [17]. Moreover, Shamsi-Goushk et al. showed that nano-curcumin supplementation not only is an effective agent in lowering blood lipids and increasing HDL but also its lipid-improving effects are significantly higher than those of curcumin [18]. Despite these promising results, there is still no clear consensus of the effects of nano-curcumin supplementation on CVD risk factors, and we are aware of no prior investigation summarizing findings on this topic. To address this knowledge gap, we conducted a comprehensive systematic review and meta-analysis of randomized controlled trials (RCTs) to evaluate the effects of nano-curcumin supplementation on risk factors for CVD. 

## 2. Materials and Methods

This study was performed based on the Preferred Reporting Items for Systematic Reviews and Meta-Analyses (PRISMA) protocol for reporting systematic reviews and meta-analyses [19].

### 2.1. Search Strategy

In order to find interrelated articles, we performed a comprehensive literature search in the online databases, including PubMed, Scopus, Embase, and ISI web of science, up to May 2021. The following MeSH and non-MeSH terms were used in our search strategy: (“nano-curcumin”(Title/Abstract) OR “nano curcumin”(Title/Abstract)) AND (Intervention OR “Intervention Study”(Title/Abstract) OR “Intervention Studies”(Title/Abstract) OR “controlled trial”(Title/Abstract) OR randomized OR randomized OR random OR randomly OR placebo OR “clinical trial”(Title/Abstract) OR Trial OR “randomized controlled trial”(Title/Abstract) OR “randomized clinical trial”(Title/Abstract) OR RCT OR blinded OR “double blind”(Title/Abstract) OR “double blinded”(Title/Abstract) OR trial OR “clinical trial”(Title/Abstract) OR trials OR “Pragmatic Clinical Trial”(Title/Abstract) OR “Cross-Over Studies”(Title/Abstract) OR “Cross-Over”(Title/Abstract) OR “Cross-Over Study”(Title/Abstract) OR parallel OR “parallel study”(Title/Abstract) OR “parallel trial”(Title/Abstract)). There were no time and language restrictions. Moreover, the bibliography of all relevant papers was checked to find potential missing publications. All searched studies were included in the Endnote software for screening, and subsequently, duplicate citations, and unpublished studies were removed.

### 2.2. Inclusion Criteria

In our study, we considered trials that met the following criteria: (1) placebo-controlled RCTs, (2) studies that were performed on adults (≥18 years), (3) used a nano-curcumin supplementation/consumption intervention, (4) RCTs with an intervention length of at least two weeks, (5) studies that assessed risk factors for CVD (lipid profile, glycemic control factors, inflammatory mediators, obesity measures, blood pressure (BP)) as an outcome, (6) If >1 article was published from a single dataset, the more thorough article was incorporated. RTCs containing > 1 intervention group were deemed independent datasets.

### 2.3. Exclusion Criteria

Exclusion criteria were as follows: (1) studies with cohort, cross-sectional, and case-control design, (2) review articles, (3) ecological studies, (4) investigations using any intervention (e.g., exercise) in their control group, (5) studies without a placebo or control group and those which were not randomized and/or were performed on offspring or teenagers, and (6) studies using nano-curcumin in combination with other nutritional compounds.

### 2.4. Data Extraction

Two independent investigators (OA and MRK) completed data extraction from each of the qualified RCTs. Extracted data contained the name of the first author, publication year, location of the study, study design, sample size in each group, individuals’ characteristics (e.g., mean age, sex, and BMI), duration of the intervention, the nano-curcumin dose utilized, mean changes and standard deviations (SDs) of cardiovascular risk factors throughout the trial for both the intervention and placebo groups, as well as the confounding variables adjusted in the analyses. If data were reported in different units, we converted them to the conventional units of an expression.

### 2.5. Quality Assessment

The quality of qualified studies was measured by two independent reviewers (OA and MRK) by using the Cochrane Collaboration modified risk of bias tool, in which the risk of bias in RCTs is assessed in seven domains, including random sequence generation, allocation concealment, reporting bias, performance bias, detection bias, attrition bias, and other sources of bias [20]. Consequently, the terms “Low,” “High,” or “Unclear” were used to judge each domain. Furthermore, any dissimilarity between reviewers was resolved by the corresponding author.

### 2.6. Statistical Analysis

In this study, weighted mean differences (WMD) and their standard deviation (SD) of CVD risk factor measures from both the intervention and control groups were extracted and utilized to find the overall effect sizes as established by the random-effects model following the DerSimonian and Laird method [21]. Additionally, when mean changes were not reported, we calculated them by using this formula: mean change = final values − baseline values, while SD changes were calculated by the following formula [22]:

(1) SD change=(SD baseline)^2+(SD final)^2−(2R × SD baseline × SD)

If outcome variables were reported in different units, we converted them to the conventional units of expression through existing suitable formulas. Moreover, we converted standard errors (SEs), 95% confidence intervals (CIs), and interquartile ranges (IQRs) to SDs using the method of Hozo et al. [23]. We applied a random-effects model, which takes between-study variations into account to find the overall effect size. Furthermore, we tested between-study heterogeneity by Cochran’s Q test and measured this by the I-square (I^2^) statistic [24]. An I^2^ > 40% or *p* < 0.05 was considered high between-study heterogeneity and subgroup analysis was used to detect potential sources of heterogeneity [25]. Subgroup analyses were performed according to pre-planned criteria, including study duration (<12 and ≥12 weeks), baseline serum concentrations of lipid profile, glycemic indices, systolic blood pressure (SBP), diastolic blood pressure (DBP), CRP, Health status (type 2 diabetes, metabolic syndrome, migraine (as inflammation contributes to its pathogenesis), and other conditions) and BMI baseline (overweight (25–29.9 kg·m^−2^) and obese (>30 kg·m^−2^)). We accomplished sensitivity analysis to find the effect of each particular study on the overall estimation [26]. The possibility of publication bias was checked through the Egger’s regression test and by visual inspection of funnel plot tests [27]. Statistical analysis was carried out using STATA, version 11.2 (Stata Corp, College Station, TX, USA). In all analyses, the *p*-values < 0.05 were considered statistically significant.

### 2.7. Certainty Assessment

The overall certainty of evidence across RCTs was rated using the Grading of Recommendations Assessment, Development, and Evaluation (GRADE) Working Group guidelines. According to the corresponding evaluation criteria, the quality of evidence was classified into four categories: high, moderate, low, and very low [28].

## 3. Results

The databases’ primary search generated 166 studies, 76 of which were duplicates that were consequently removed. Another 81 studies were excluded for the following reasoning: irrelevant studies based on title and abstracts (*n* = 62), animal (*n* = 17), and review studies (*n* = 2). Finally, nine RCTs attaining all needed criteria were included for meta-analysis in the current investigation (Supplementary Figure S1).

### 3.1. Study Characteristics

The detailed characteristics of the nine RCTs [14,29,30,31,32,33,34,35,36], including 11 intervention arms, are summarized in Table 1. There were 510 participants included (cases = 269 and control = 241) in these RCTs dated between 2016 and 2021. All studies were designed as parallel studies. RCTs’ lengths ranged from between 6 and 12 weeks, with sample sizes ranging from 16 to 84 participants. Participants’ demographic characteristics included ages ranging from 35 to 62 years, while baseline BMI varied between 26.1 and 32.22 kg·m^−2^. Participants were patients with T2DM, migraine, metabolic syndrome, nonalcoholic fatty liver disease, and hemodialysis. Eight RCTs included both sexes, and one study women participants only [33]. In seven RCTs, the dose of nano-curcumin was 80 mg/d, while Jazayeri-Tehrani et al. [32] and Vafadar-afshar et al. used 40 and 120 mg/d [35], respectively. Quality assessment characteristics of studies are provided in Supplementary Table S1.

### 3.2. Meta-Analysis

#### 3.2.1. The Effects of Nano-Curcumin Supplementation on Triglyceride (TG)

**Figure 1:**
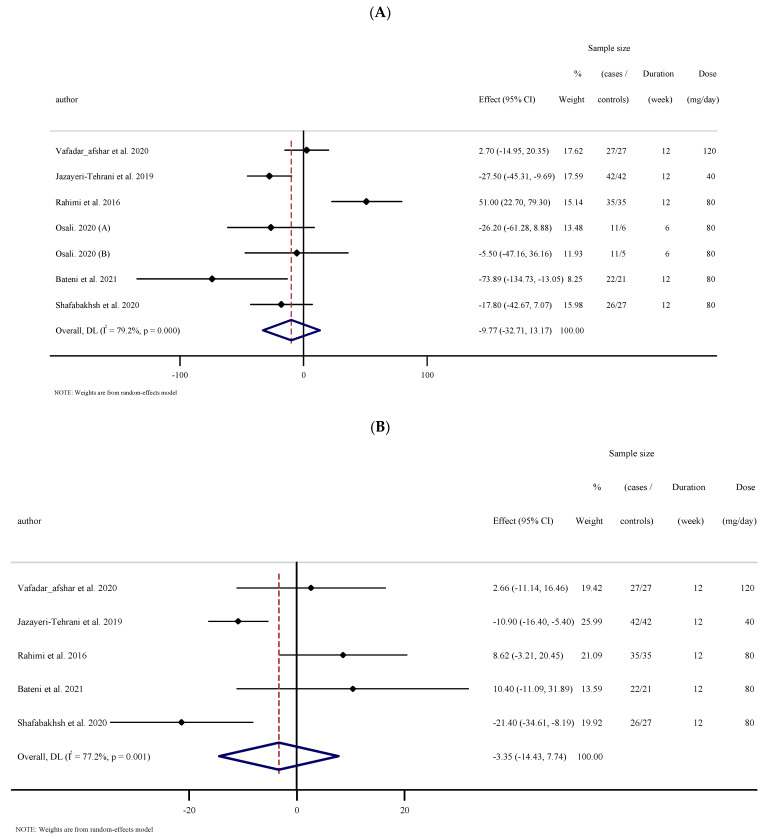

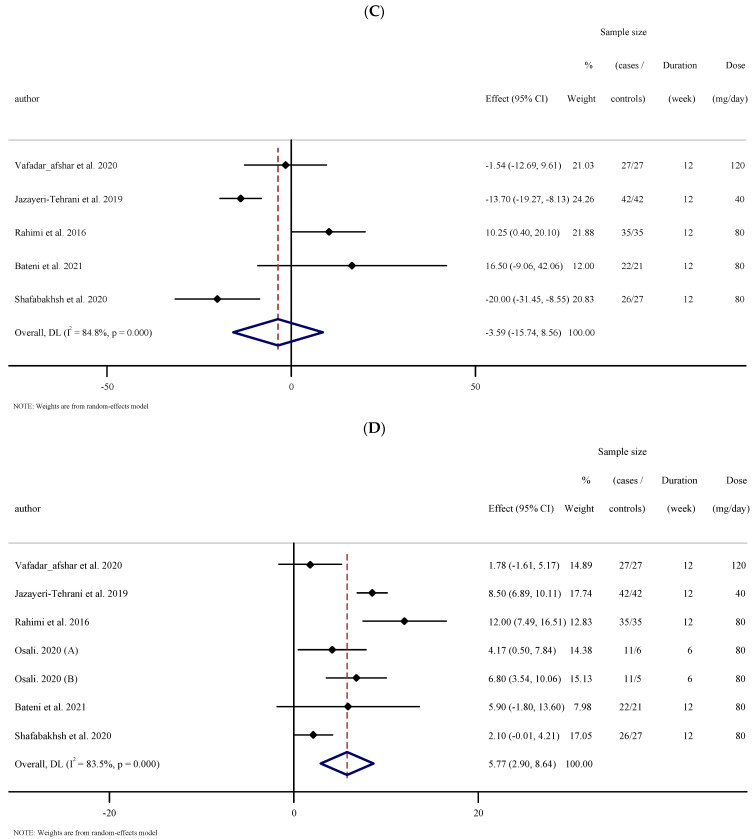
Forest plot of the random-effects meta-analysis of the effect of nano-curcumin on (**A**) TG, (**B**) TC, (**C**) LDL and cholesterol, (**D**) HDL cholesterol. (**A**) Forest plot of the random-effects meta-analysis of the effect of nano-curcumin on TG. (**B**). Forest plot of the random-effects meta-analysis of the effect of nano-curcumin on TC. (**C**). Forest plot of the random-effects meta-analysis of the effect of nano-curcumin on LDL. (**D**). Forest plot of the random-effects meta-analysis of the effect of nano-curcumin on HDL.

The results for the analysis of six studies, with seven effect sizes that measured TG concentrations following nano-curcumin supplementation (174 cases and 163 controls) indicated no significant reduction in TG concentrations (WMD: −9.76 mg/dL; 95% CI: −32.71 to 13.17; *p* = 0.404), with high heterogeneity between studies ((I^2^ = 79.2%, *p* < 0.001); (Figure 1A)). Subgroup analysis was performed to determine the heterogeneity sources. The results of subgroup analysis showed that nano-curcumin supplementation significantly reduced TG concentrations in participants who had baseline TG ≥ 150 mg/dL and in obese individuals with BMI > 30 kg·m^−2^ (Table 2).

#### 3.2.2. The Effects of Nano-Curcumin Supplementation on Total Cholesterol (TC)

Based on the outcomes of five RCTs (152 cases and 152 controls), there was no significant change in TC concentrations following nano-curcumin supplementation (WMD: −3.34 mg/dL; 95% CI: −14.43 to 7.73; *p* = 0.554), with high between-study heterogeneity ((I^2^ = 72.2%, *p* = 0.001); (Figure 1B)). Subgroup analysis showed that nano-curcumin supplementation significantly reduced TC concentrations when baseline TC ≥ 200 mg/dL or the intervention was performed in obese individuals with BMI > 30 kg·m^−2^ (Table 2).

#### 3.2.3. The Effects of Nano-Curcumin Supplementation on LDL-C

Overall results from five RCTs (152 cases and 152 controls), did not reveal significant alterations in LDL cholesterol concentrations (WMD: −3.59 mg/dL; 95% CI: −15.74 to 8.56; *p* = 0.562), with high between-study heterogeneity ((I^2^ = 84.8%, *p* < 0.001); (Figure 1C)). Subgroup analyses demonstrated nano-curcumin supplementation significantly altered LDL-C in participants with baseline LDL-C ≥ 100 (mg/dL) and BMI > 30 kg·m^−2^ (Table 2).

#### 3.2.4. The Effects of Nano-Curcumin Supplementation on HDL-C

Pooled effect sizes from six studies containing seven arms (174 cases and 163 controls) revealed a significant change in HDL cholesterol concentrations (WMD: 5.77 mg/dL; 95% CI: 2.90 to 8.64; *p* < 0.001, I^2^ = 83.5%, *p* < 0.001) following nano-curcumin supplementation (Figure 1D). Subgroup analyses established significant changes regardless of baseline HDL-C (<40 and ≥40 mg/dL) and trial duration (<12 and ≥12 weeks), and in those with metabolic syndrome, overweight (25–29.9 kg·m^−2^) and with obesity (>30 kg/m^2^) (Table 2).

#### 3.2.5. The Effects of Nano-Curcumin Supplementation on Fasting Blood Sugar (FBS)

**Figure 2:**
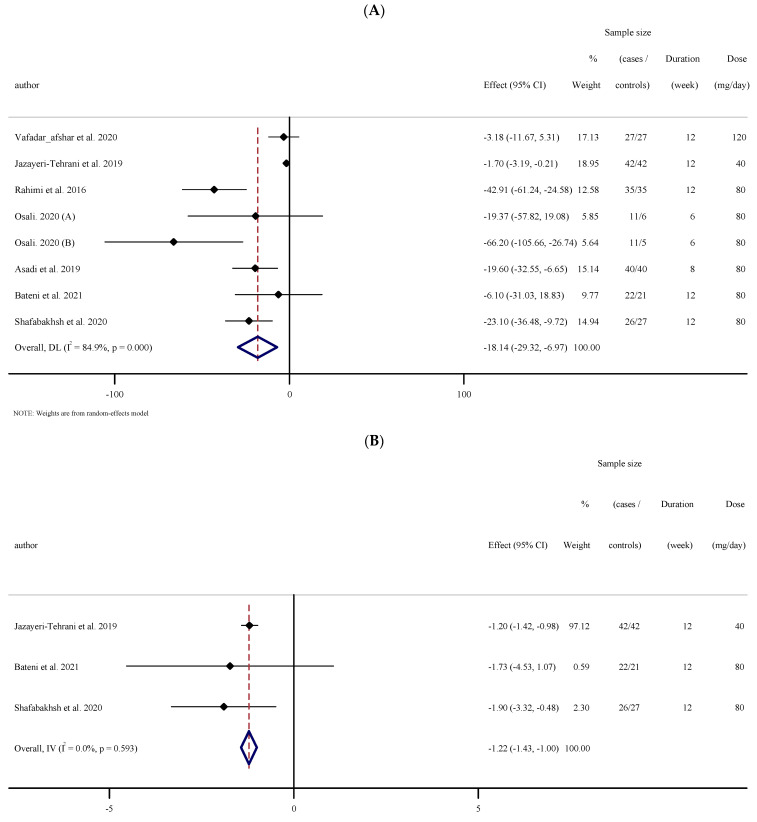

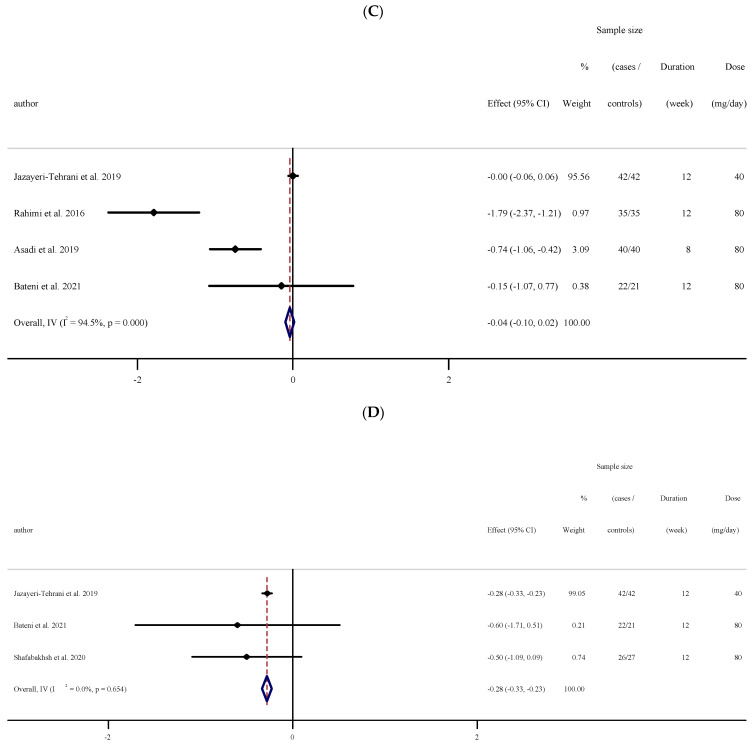
Forest plot of the random-effects meta-analysis of the effect of nano-curcumin on (**A**) FBS, (**B**) fasting insulin, (**C**) HbA1c, and (**D**) HOMA-IR. (**A**). Forest plot of the random-effects meta-analysis of the effect of nano-curcumin on FBS. (**B**). Forest plot of the random-effects meta-analysis of the effect of nano-curcumin on fasting insulin. (**C**). Forest plot of the random-effects meta-analysis of the effect of nano-curcumin on HbA1c. (**D**). Forest plot of the random-effects meta-analysis of the effect of nano-curcumin on HOMA-IR.

The outcomes analysis of the seven studies with eight effect sizes that measured FBS concentrations subsequent to nano-curcumin supplementation (210 cases and 200 controls) indicated an overall effect of a significant reduction in FBS concentrations ((WMD: −18.14 mg/dL; 95% CI: −29.31 to −6.97; *p* = 0.001, I^2^ = 84.9%, *p* < 0.001); (Figure 2A)). Subgroup analyses established significant changes when baseline FBS was <100 or ≥100 (mg/dL), trial duration was <12 and ≥12 weeks, and in type 2 diabetic and overweight individuals (25–29.9 kg·m^−2^) (Table 2).

#### 3.2.6. The Effects of Nano-Curcumin Supplementation on Fasting Insulin, Homeostatic Model Assessment for Insulin Resistance (HOMA-IR), and Hemoglobin A1c (HbA1c)

Overall results from three investigations (90 cases and 80 controls) showed a significant change in fasting insulin concentrations (WMD: −1.21 mg/dL; 95% CI: −1.43 to −1.00; *p* < 0.001, I^2^ = 0.0%, *p* = 0.593) and HOMA-IR (WMD: −0.28 mg/dL; 95% CI: −0.33 to −0.23; *p* < 0.001, I^2^ = 0.0%, *p* = 0.654). However, the results of four studies did not indicate significant changes in HbA1c (WMD: −0.66 mg/dL; 95% CI: −1.41, 0.08; *p* = 0.081, I^2^ = 94.5%, *p* < 0.001) (Figure 2B–D).

#### 3.2.7. The Effects of Nano-Curcumin Supplementation on BP

**Figure 3:**
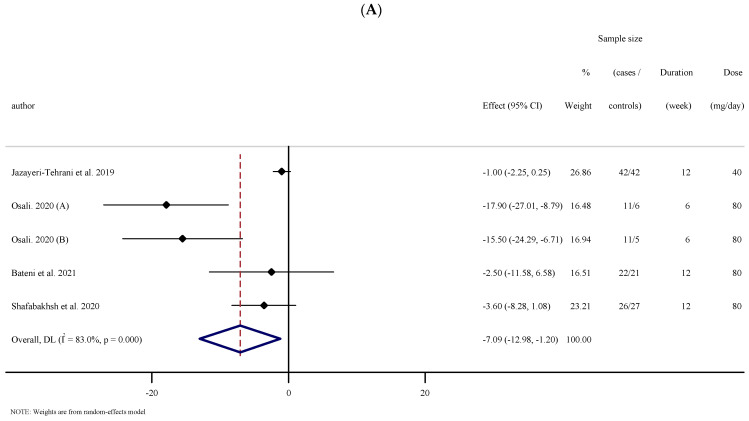

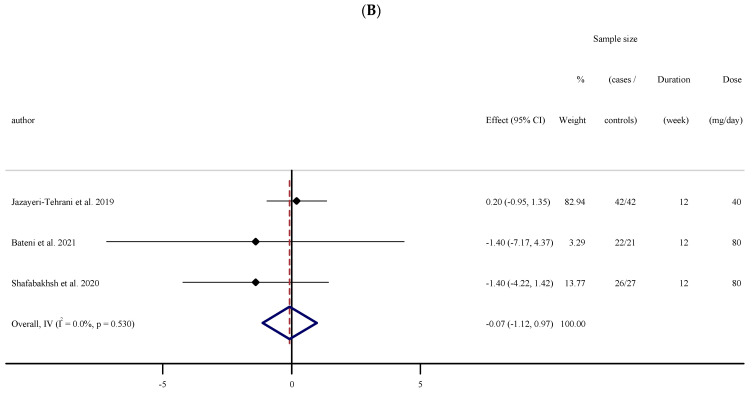
Forest plot of the random-effects meta-analysis of the effect of nano-curcumin on (**A**) SBP and (**B**) DBP. (**A**). Forest plot of the random-effects meta-analysis of the effect of nano-curcumin on SBP. (**B**). Forest plot of the random-effects meta-analysis of the effect of nano-curcumin on DBP.

Four eligible RTCs with five treatment arms, including 203 participants, examined the effect of nano-curcumin supplementation on SBP. Combining their findings based on the random-effects model, we found that SBP was significantly reduced after the intervention (WMD: −2.50 mg/dL; 95% CI: −11.58, 6.58; *p* = 0.590, I^2^ = 83.0%, *p* = 0.018); (Figure 3A). Subgroup analysis showed that the results remained significant when baseline SBP was ≥120 mmHg, trial duration was <12 weeks, and when the intervention was performed in individuals with metabolic syndrome (Table 2). Pooling effect sizes from three publications including 170 participants, disclosed that nano-curcumin supplementation had no significant effect on DBP compared with the placebo (WMD: −0.07 mg/dL; 95% CI: −1.12 to 0.97; *p* = 0.891, I^2^= 0.0%, *p* = 0.530) (Figure 3B).

#### 3.2.8. The Effects of Nano-Curcumin Supplementation on CRP

**Figure 4:**
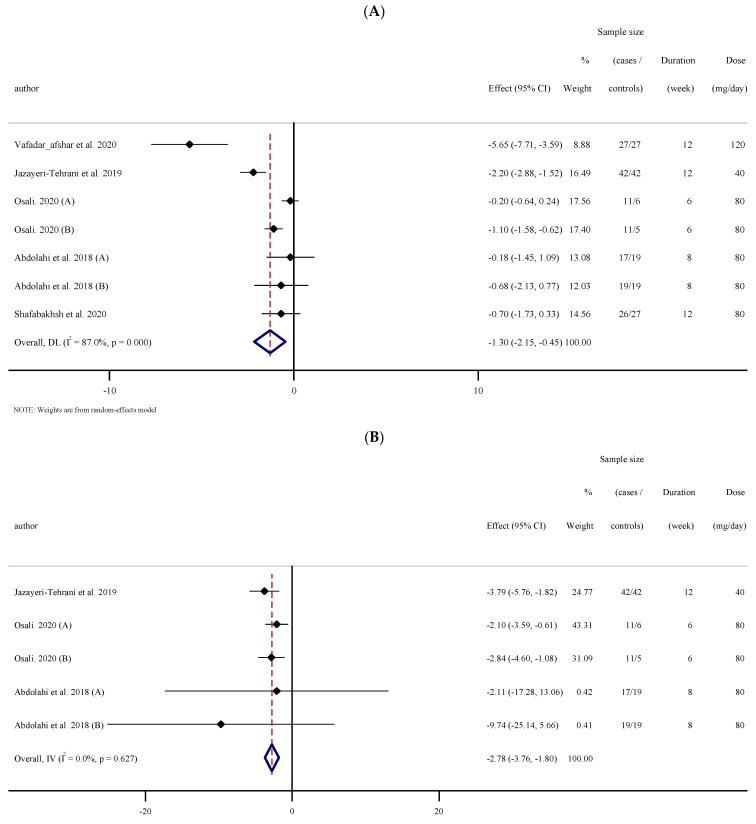

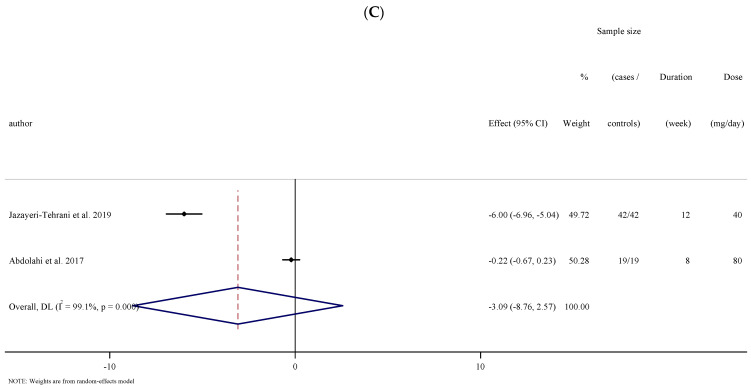
Forest plot of the random-effects meta-analysis of the effect of nano-curcumin on (**A**) CRP, (**B**) IL-6, (**C**) TNF-α. (**A**). Forest plot of the random-effects meta-analysis of the effect of nano-curcumin on CRP. (**B**). Forest plot of the random-effects meta-analysis of the effect of nano-curcumin on IL-6. (**C**). Forest plot of the random-effects meta-analysis of the effect of nano-curcumin on TNF-α.

Pooled results from five studies with seven effect sizes showed a significant reduction in serum concentrations of CRP compared with the placebo (WMD: −1.29 mg/L; 95% CI: −2.15 to −0.44; *p* = 0.003, I^2^ = 87.0%, *p* < 0.001); (Figure 4A). Studies conducted on those with baseline CRP ≥ 3 (mg/L), BMI 25–29.9 kg·m^−2^, a trial duration ≥12 weeks, and for people in the other group in terms of their health status, revealed a greater reduction in serum concentrations of CRP (Table 2).

#### 3.2.9. The Effects of Nano-Curcumin Supplementation on IL-6 and TNF-α

Pooled effect sizes from three studies containing five arms (100 cases and 73 controls) showed a significant reduction in serum concentrations of IL-6 (WMD: −2.78 mg/dL; 95% CI: −3.76 to −1.79; *p* < 0.001, I^2^ = 0.0%, *p* = 0.627); (Figure 4B). However, the analysis of the results of two studies [29] did not show a significant reduction in serum concentrations of TNF-α (WMD: −3.09 mg/dL; 95% CI: −8.75 to 2.57; *p* = 0.284, I^2^ = 99.1%, *p* < 0.001); (Figure 4C).

#### 3.2.10. The Effects of Nano-Curcumin Supplementation on the Anthropometric Indices

The results of the analysis of five studies for body mass, six studies for BMI, three studies for fat mass (FM) and four studies for waist circumference (WC), did not show a significant effect of nano-curcumin supplementation on anthropometric indices including body mass (WMD: −0.51 mg/dL; 95% CI: −1.85 to 0.82; *p* = 0.449, I^2^ = 0.0%, *p* = 0.974), BMI (WMD: −0.35 mg/dL; 95% CI: −0.76 to 0.04; *p* = 0.079, I^2^ = 29.0%, *p* = 0.207), FM (WMD: −0.86 mg/dL; 95% CI: −1.95 to 0.23; *p* = 0.123, I^2^ = 4.3%, *p* = 0.371) and WC (WMD: −1.32 mg/dL; 95% CI: −3.89 to 1.23; *p* = 0.310, I^2^ = 67.4%, *p* = 0.015) (Supplementary Figures S2–S5).

### 3.3. Publication Bias

According to Egger’s regression test, there was no evidence of publication bias for studies examining the effect of nano-curcumin supplementation on TG (*p* = 0.789), TC (*p* = 0.365), LDL (*p* = 0.352), HDL (*p* = 0.816), fasting insulin (*p* = 0.299), HOMA-IR (*p* = 0.094), HbA1c (*p*=0.193), SBP (*p*=0.081), DBP (*p*=0.330), CRP(*p*=0.328), IL-6 (*p* = 0.416), BMI (*p* = 0.838), WC (*p* = 0.083), and FM (*p* = 0.570). However, there was significant publication bias for FBS (*p*=0.013) and body mass (*p* = 0.010), which was visually confirmed after funnel plot analysis (Supplementary Figures S6–S22).

### 3.4. Sensitivity Analysis

To explore each study’s impact on the overall effect size, we omitted each trial from the analysis step by step. After removing the study by Rahimi et al., 2016, Abdolahi et al., 2017, and Jazayeri-Tehrani et al., 2019, respectively, the overall result of TG (WMD: −18.12 mg/dL, 95% CI: −34.14 to −2.11), TNF-α (WMD: −6, 95% CI: −6.95 to −5.04) and HbA1C (WMD: −0.93 mg/L, 95% CI: −1.76 to −0.11), BMI (WMD: −0.54, 95% CI: −1.06 to −0.19), and FM (WMD: −1.63, 95% CI: −3.18 to −0.08) were significantly changed. In addition, eliminating the study by Osali et al., 2020 (A, B) significantly changed the overall result of SBP (WMD: −4.48, 95% CI: −9.39 to 0.41) and (WMD: −5.06, 95% CI: −10.56 to 0.42).

### 3.5. Grading of Evidence

To assess the quality of evidence for outcomes, the GRADE framework was performed and determined the effect of fasting insulin, HOMA-IR, and IL-6 to be of high quality. The evidence about HDL-C, SBP, CRP, body mass, WC, FM, BMI, and DBP was downgraded to moderate. According to the GRADE protocol, evidence regarding TG, TC, LDL, FBS, HbA1c, and TNF-α was identified as very low quality (Table 3).

## 4. Discussion

In this meta-analysis, we evaluated the effects of nano-curcumin supplementation on risk factors for CVD, including lipid and glycemic profiles, BP, inflammatory markers, and body composition. According to the results of this study, nano-curcumin supplementation was associated with improvements in the glycemic profile by decreasing FBG, fasting insulin, and HOMA-IR. Interestingly, nano-curcumin supplementation resulted in increases in HDL concentrations. In terms of other lipid profile markers (TG, TC, and LDL), subgroup analyses showed that nano-curcumin supplementation had more favorable effects on lipid profiles in individuals with dyslipidemia at baseline (TG > 150 mg/dL; TC > 200 mg/dL, and LDL cholesterol > 100 mg/dL). Nano-curcumin supplementation also showed favorable anti-inflammatory effects by decreasing CRP and IL-6. Moreover, our results demonstrated a decline in SBP levels, highlighting the hypotensive effects of nano-curcumin supplementation. However, nano-curcumin had no significant effects on body composition indices.

In recent decades, the health benefits and cardioprotective effects of curcumin and its nanorange formulations, namely nano-curcumin have been rigorously reported [9,11,37]. Some epidemiological surveys showed the positive effects of curcumin supplementation on the risk of different chronic diseases [38,39,40]. However, no epidemiological study evaluated the association of long-term nano-curcumin intake with CVD risk factors. Both curcumin and nano-curcumin have the same chemical structure. Since nano-curcumin has higher bioavailability and solubility than curcumin (which increases its circulation and retention in the body and overcomes the physiological barriers of curcumin), it is hypothesized that nano-curcumin more effectively improves CVD risk. However, there are limited data to support this hypothesis. For the first time, our research showed a significant cardioprotective effect of nano-curcumin supplementation via a systematic review and meta-analysis.

Numerous epidemiological studies have reported that dyslipidemia is associated with increased CVD risk [41,42,43]. Current recommendations based on the American Diabetes Association (ADA), the European Association for Cardiovascular Prevention and Rehabilitation (EACPR), and the National Cholesterol Education Program (NCEP) guidelines, are to use lipid-lowering agents to prevent CVD in patients with dyslipidemia [44,45,46]. These hypolipidemic agents have been shown to decrease the risk of cardiovascular events [47,48,49,50]. Our findings showed that nano-curcumin supplementation might act as lipid-lowering agents by reducing TG, TC, and LDL in patients with dyslipidemia. Interestingly, based on our analysis, nano-curcumin supplementation may have HDL-increasing effects. Many prospective studies from different racial and ethnic groups worldwide have confirmed that HDL is a strong, consistent, and independent predictor of the occurrence of cardiovascular events (myocardial infarction, ischemic stroke) [51,52,53]. The previous meta-analysis revealed that HDL is scarcely affected by dietary supplement interventions and these studies failed to see any significant improvements in HDL concentrations [54,55]. Therefore, the positive effects of nano-curcumin supplementation on HDL concentrations can significantly benefit this compound as a dietary supplement. Indeed, the lipid-improving effect of nano-curcumin supplementation has been reported in both animal and human studies. For example, Reda et al. evaluated the response of quails to dietary nano-curcumin levels on the lipid profile [56]. The authors reported that nano-curcumin supplementation could improve the lipid profile by increasing HDL and decreasing TG and TC. Moreover, Shamsi-Goushk et al. showed that nano-curcumin is not only an effective agent in lowering blood lipids and increasing HDL but also its lipid-improving effects are significantly higher than those of curcumin [18].

Although previous meta-analytic work highlighted the beneficial effects of curcumin supplementation on glycemic control, [57] our meta-analysis is the first to report the beneficial impact of nano-curcumin on this matter. Several animal studies demonstrated that nano-curcumin supplementation improves glycemic control in diabetic rats. For example, Gouda et al. showed that three weeks of nano-curcumin supplementation decreased FBG while it increased fasting insulin, gene expression of insulin, and insulin receptors over controls [58]. Moreover, Shamsi-Goushki et al. compared the effects of curcumin and nano-curcumin supplementation on the glycemic profile of type 2 diabetic rats [18]. According to their findings, FBS and insulin resistance were significantly decreased in diabetic rats following curcumin and nano-curcumin supplementation. However, there was no significant difference between the curcumin and nano-curcumin supplementation groups. The study by Ganugula et al. showed that nano-curcumin supplementation, but not curcumin, significantly decreased blood glucose in STZ-induced diabetic rats [59]. Furthermore, their TUNEL assay data suggested that nano-curcumin supplementation offered sounder protection than plain curcumin to prevent STZ induced beta-cell death. The hypoglycemic effects of nano-curcumin supplementation have also been observed in human studies. From all seven studies that evaluated nano-curcumin supplementation effects on the glycemic profile in our analysis, six studies [14,31,32,33,34,35] showed significant improvements in the glycemic profile. From all included studies, only one study [36] reported no significant differences in the glycemic profile, including FBS, HbA1c, HOMA-IR, and HOMA-β following nano-curcumin supplementation compared to the placebo. These findings suggest the effectiveness of nano-curcumin supplementation in improving the glycemic profile.

Inflammation plays an important role in all stages of CVD, from the initial lesion to end-stage thrombotic complications [60]. Inflammatory biomarkers are becoming important additions in defining those at the greatest risk of progressive vascular disease [61]. For example, it has been reported that CRP, IL-6, and TNF-α are predictors of incident coronary and cardiovascular events and total mortality [62]. Extensive research has shown that curcumin has solid anti-inflammatory activities [63,64,65]. In vitro and in vivo studies, especially clinical trials, indicate nano-curcumin, an anti-inflammatory agent, may be a potential therapeutic intervention in some chronic inflammatory diseases such as cancer, multiple sclerosis, asthma, coeliac disease, and inflammatory bowel disease [15,66,67,68]. Some human studies confirmed the inflammatory modulating effects of nano-curcumin by reducing CRP [32,33,35], IL-6 [32,33], and TNF-α [32]. Our findings underlined the anti-inflammatory properties of nano-curcumin supplementation by reducing CRP and IL-6. However, TNF-α concentrations did not change following nano-curcumin supplementation which might be due to the low number of included studies.

Hypertension is one of the chief risk factors for CVD [69]. It has been revealed that SBP is a stronger predictor of events than diastolic BP [70,71], and therefore, reducing SBP results significantly declines the CVD risk. Previous meta-analytic work failed to find the effectiveness of curcumin supplementation on BP [72,73]. However, our findings indicate that nano-curcumin may significantly improve SBP. The reasoning behind the beneficial BP-lowering effect of nano-curcumin compared to the lack of improvement following curcumin supplementation is unclear. However, nano-curcumin’s higher solubility and bioavailability compared to curcumin may be a potential factor [74]. Further research is warranted to directly compare BP responses to nano-curcumin and curcumin supplementation, especially in hypertensive cohorts.

The cardiovascular protective effects of nano-curcumin supplementation may be attributed to several potential mechanisms. Firstly, nano-curcumin has anti-inflammatory properties. Nano-curcumin may attenuate the metabolism of prostaglandins and lipoxygenases, which are involved in the appearance of inflammatory signs and lead to the production of free radicals [75]. Animal experiments showed that nano-curcumin supplementation reduced inflammation by inhibiting nuclear factor-*kappa* B (NF-kB) activation [76,77]. The NF-kB pathway is a potent immunostimulatory pathway, and its suppression results in decreasing pro-inflammatory cytokine concentrations such as IL-6. Since IL-6 can induce CRP gene expression, inhibiting the NF-kB/IL-6 pathway decreases CRP concentrations [78]. Secondly, nano-curcumin supplementation may have cardioprotective effects by boosting antioxidant defenses. It has been shown that curcumin and nano-curcumin supplementation exhibits antioxidant properties [79]. Potphode et al. showed that adding nano-curcumin to curcumin can enhance the antioxidant capacity in diabetic mice by increasing Superoxide dismutase (SOD), Catalase (CAT), and Glutathione peroxidase (GPx) activity when compared to both a control and curcumin alone group [80]. These findings suggest that nano-curcumin supplementation may enhance the antioxidant effects of curcumin. Indeed, dietary supplementations with antioxidant properties have been shown to modulate lipid profiles by improving oxidative stress [81]. Therefore, the antioxidant and anti-inflammatory properties of nano-curcumin may be partially responsible for its hypolipidemic effects. Fourthly, curcumin supplementation significantly elevated apo-AI, a major component of HDL [82], 17%, and consequently, the apo-AI/apoB ratio in HFD-fed hamsters [83]. Furthermore, another study showed a striking decrease in the mean apoB/apo-AI ratio after the 30-day curcuma supplementation in healthy participants [84]. This increase in serum apoA-I coincides with increases in HDL [85]. Fifthly, a large number of studies showed that curcumin supplementation could improve insulin sensitivity. The hypoglycemic effects of nano-curcumin may be because of its adiponectin enhancing, antioxidant properties, and anti-inflammatory effects [86,87]. Finally, hypotensive effects of nano-curcumin supplementation may be exhibited by an improved Na+, K+-ATPase activity [88], as this enzyme is an essential mediator of vasorelaxation [89,90]. Nano-curcumin supplementation may also inhibit the angiotensin-converting enzyme (ACE), thereby reducing angiotensin-II-induced oxidative stress and indirectly enhancing NO production [91]. Oxidative stress through the disruption of NO production leads to oxidative damage of the endothelial cells and ultimately endothelial dysfunction [92]. Nano-curcumin supplementation enhances NO synthesis and bioavailability by reducing oxidative stress and improving endothelium-dependent vasodilation and reducing protein, lipid, and DNA damage [91,93]. Furthermore, nano-curcumin supplementation cleanses reactive oxygen and nitrogen species, and enhances antioxidant enzymes such as SOD, CAT, and GPx [80]; thereby protecting endothelial cells from oxidative damage and regulating BP [94,95]. Moreover, the suppression of Nf-kB by its anti-inflammatory activity might be another possible mechanism for the hypotensive effects of this compound [76,77].

There are some limitations to the current work that should be considered when deciphering our results. First, since all but one trial lasted less than three months, our analysis is unable to show the long-term effects of nano-curcumin supplementation on risk factors for CVD. Second, there was a notable heterogeneity between studies, resulting from differences between participants’ characteristics, and nano-curcumin dosage used in the included studies. Thirdly, the relatively low number of participants in the included studies is another limitation of our meta-analysis. Finally, all included studies were conducted in one country (Iran), and consequently, further research in different countries is needed before our positive findings can be assured in other ethnic cohorts.

## 5. Conclusions

In conclusion, nano-curcumin supplementation was associated with an improved glycemic profile by decreasing FBG, fasting insulin, and HOMA-IR. Moreover, nano-curcumin supplementation resulted in a rise of HDL. The hypolipidemic effects (TG, TC, and LDL decrements) of this compound were demonstrated in patients with dyslipidemia (TG > 150mg/dL; TC > 200 mg/dL; and LDL cholesterol > 100 mg/dL). We also found decreases in CRP, IL-6, and SBP, which show the favorable anti-inflammatory and hypotensive effects of nano-curcumin supplementation.

## Figures and Tables

**Table 1:** Characteristic of included studies.

Studies	Country	Study Design	Participant	Sample Size and Sex	Sample Size		Trial Duration (Week)	Means Age		Means BMI		Intervention	
					IG	CG		IG	CG	IG	CG	Intervention Dose (mg/d)	Control Group
**Vafadar_afshar et al., 2020**	**Iran**	**Parallel = l, R, PC, DB**	**Hemodialysis Patients**	**M/F (F:20, M:34)**	27	27	12	55.33 ± 12.95	59.05 ± 7.68	26.1 ± 5.19	27.19 ± 5.19	120	placebo
**Jazayeri-Tehrani et al., 2019**	Iran	parallel, R, PC, DB	non-alcoholic fatty liver disease	M/F (F:38, M:46)	42	42	12	41.8 ± 5.6	42.5 ± 6.2	30.6 ± 2.14	30.7 ± 2.35	40	placebo
**Abdolahi et al., 2017**	Iran	parallel, R, PC, DB	migraine patients	M/F (F:30, M:8)	19	19	8	37.36 ± 8.49	36.57 ± 8.15	27.59 ± 4.57	26.94 ± 3.87	80	placebo
**Rahimi et al., 2016**	Iran	parallel, R, PC, DB	type 2 diabetic	M/F (F:39, M:31)	35	35	12	56.34 ± 11.17	60.95 ±1 0.77	26.92 ± 2.71	27.27 ± 3.59	80	placebo
**Osali. 2020 (A)**	Iran	parallel, R, PC, DB	metabolic syndrome	F:22	11	6	6	62.3 ± 1.23	62.3 ± 1.23	31.24 ± 3.12	32.22 ± 2.46	80	placebo
**Osali. 2020 (B)**	Iran	parallel, R, PC, DB	metabolic syndrome	F:22	11	5	6	62.3 ± 1.23	62.3 ± 1.23	29.54 ± 2.67	29.02 ± 1.56	80	placebo
**Asadi et al., 2019**	Iran	parallel, R, PC, DB	type 2 diabetes	M/F (F:70, M:10)	40	40	8	53.3 ± 6.5	54.6 ± 6.2	31.1 ± 4.2	30.8 ± 3.8	80	placebo
**Bateni et al., 2021**	Iran	parallel, R, PC, DB	metabolic syndrome	M/F (F:33, M:10)	22	21	12	50 ± 9	54 ± 7	29.9 ± 4.3	29.4 ± 4.5	80	placebo
**Abdolahi et al., 2018 (A)**	Iran	parallel, R, PC, DB	Migraine	M/F (F:29, M:7)	17	10	8	35.82 ± 8.2	36.15 ± 8.67	26.02 ± 4.04	26.16 ± 4.27	80	placebo
**Abdolahi et al., 2018 (B)**	Iran	parallel, R, PC, DB	Migraine	M/F (F:30, M:8)	19	9	8	37.36 ± 8.5	36.57 ± 8.15	27.59 ± 4.57	26.94 ± 3.87	80	placebo
**Shafabakhsh et al., 2020**	Iran	parallel, R, PC, DB	Diabetes on Hemodialysis	M/F (F:21, M:32)	26	27	12	58.3 ± 9.4	56.2 ± 9.8	27.9 ± 4.9	27.1 ± 4.2	80	placebo

Abbreviations: IG, intervention group; CG, control group; DB, double-blinded; PC, placebo-controlled; CO, controlled; RA, randomized; NR, not reported; F, Female; M, Male.

**Table 2:** Subgroup analyses of nano-curcumin consumption on cardiovascular risk factors in adults.

	No	WMD (95%CI)	P within Group	Heterogeneity			
				P Heterogeneity	I^2^	P between Subgroups	Tau-Squared
Subgroup analyses of nano-curcumin supplementation on TG.							
Overall effect	7	−9.76 (−32.71, 13.17)	0.404	<0.001	79.2%		696.43
Baseline TG (mg/dL)							
<150	2	25.53 (−21.73, 72.79)	0.290	0.005	87.6%	<0.001	1000
≥150	5	−24.87 (−37.34, −12.40)	**<0.001**	0.445	0.0%		0.0
Trial duration (week)							
<12	2	−17.61 (−44.44, 9.21)	0.198	0.456	0.0%	0.454	0.0
≥12	5	−8.05 (−37.44, 21.34)	0.591	<0.001	85.6%		893.41
Health status							
Type 2 diabetic	2	16.25 (−51.16, 83.67)	0.637	<0.001	92.2%	0.028	2200
Metabolic syndrome	3	−29.11 (−61.92, 3.68)	0.082	0.191	39.5%		334.45
Other	2	−12.37 (−41.97, 17.22)	0.412	0.018	82.1%		374.19
BMI baseline							
Overweight (25–29.9 kg/m^2^)	5	−2.74 (−32.87, 27.38)	0.858	<0.001	80.0%	0.003	874.72
Obese (>30 kg/m^2^)	2	−27.23 (−43.11, −11.35)	**0.001**	0.948	0.0%		0.0
Overall analyses of nano-curcumin supplementation on TC							
Overall effect	5	−3.34 (−14.43, 7.73)	0.554	0.001	77.2%		115.21
Baseline TC (mg/dL)							
<200	4	−0.53 (−15.66, 14.60)	0.945	0.005	77.0%	0.033	179.57
≥200	1	−10.90 (−16.40, −5.39)	**<0.001**	-	-		0.0
Health status							
Type 2 diabetic	2	−6.24 (−35.65, 23.17)	0.678	0.001	90.9%	0.185	409.66
Metabolic syndrome	1	10.40 (−11.09, 31.89)	0.343	-	-		0.0
Other	2	−5.65 (−18.59, 7.28)	0.392	0.074	68.8%		63.22
BMI baseline							
Overweight (25–29.9 kg/m^2^)	4	−0.53 (−15.66, 14.60)	0.945	0.005	77.0%	0.033	179.57
Obese (>30 kg/m^2^)	1	−10.90 (−16.40, −5.39)	**<0.001**	-	-		0.0
Subgroup analyses of nano-curcumin supplementation on LDL-C							
Overall effect	5	−3.59 (−15.74, 8.56)	0.562	<0.001	84.8%		150.46
Baseline LDL-C (mg/dL)							
<100	4	−0.14 (−15.65, 15.37)	0.986	0.001	82.8%	0.003	196.19
≥100	1	−13.70 (−19.26, −8.13)	**<0.001**	-	-		0.0
Health status							
Type 2 diabetic	2	−4.72 (−34.37, 24.91)	0.755	<0.001	93.5%	0.028	427.83
Metabolic syndrome	1	16.50 (−9.06, 42.06)	0.206	-	-		0.0
Other	2	−8.61 (−20.37, 3.13)	0.151	0.056	72.7%		53.72
BMI baseline							
Overweight (25–29.9 kg/m^2^)	4	−0.14 (−15.65, 15.37)	0.986	0.001	82.8%	0.003	196.19
Obese (>30 kg/m^2^)	1	−13.70 (−19.26, −8.13)	**<0.001**	-	-		0.0
Subgroup analyses of nano-curcumin supplementation on HDL-C							
Overall effect	7	5.77 (2.90, 8.64)	**<0.001**	<0.001	83.5%		11.41
Baseline HDL-C (mg/dL)							
<40	2	2.01 (0.21, 3.80)	**0.028**	0.875	0.0%	<0.001	0.0
≥40	5	7.61 (5.34, 9.89)	**<0.001**	0.079	52.3%		3.22
Trial duration (week)							
<12	2	5.62 (3.06, 8.18)	**<0.001**	0.293	9.5%	0.779	0.32
≥12	5	5.92 (1.93, 9.90)	**0.004**	<0.001	88.6%		16.57
Health status							
Type 2 diabetic	2	6.84 (−2.85, 16.53)	0.167	<0.001	93.4%	0.021	45.77
Metabolic syndrome	3	5.66 (3.34, 7.98)	**<0.001**	0.574	0.0%		0.0
Other	2	5.31 (−1.26, 11.88)	0.113	<0.001	91.9%		20.75
BMI baseline							
Overweight (25–29.9 kg/m^2^)	5	5.42 (1.78, 9.05)	**0.003**	0.001	79.9%	0.001	12.74
Obese (>30 kg/m^2^)	2	6.66 (2.46, 10.85)	**0.002**	0.034	77.7%		7.28
Subgroup analyses of nano-curcumin supplementation on FBS							
Overall effect	8	−18.14 (−29.31, −6.97)	**0.001**	<0.001	84.9%		170.85
Baseline FBS (mg/dL)							
<100	1	−1.70 (−3.18, −0.21)	**0.025**	-	-	<0.001	0.0
≥100	7	−22.43 (−36.02, −8.84)	**0.001**	<0.001	76.2%		220.96
Trial duration (week)							
<12	3	−31.20 (−57.78, −4.61)	**0.021**	0.087	59.1%	<0.001	330.04
≥12	5	−13.77 (−25.80, −1.73)	**0.025**	<0.001	86.2%		139.70
Health status							
Type 2 diabetic	3	−27.07 (−39.61, −14.52)	**<0.001**	0.112	54.4%	<0.001	66.60
Metabolic syndrome	3	−28.29 (−63.34, 6.76)	0.114	0.041	68.7%		655.74
Other	2	−1.74 (−3.20, −0.28)	**0.019**	0.736	0.0%		0.0
BMI baseline							
Overweight (25–29.9 kg/m^2^)	5	−24.53 (−43.50, −5.55)	**0.011**	<0.001	83.8%	<0.001	355.24
Obese (>30 kg/m^2^)	3	−10.50 (−25.81, 4.79)	0.178	0.018	75.1%		119.83
Subgroup analyses of nano-curcumin supplementation on fasting insulin							
Overall effect	3	−1.21 (−1.43, −1.00)	**<0.001**	0.593	0.0%		0.0
Subgroup analyses of nano-curcumin supplementation on hemoglubin A1c							
Overall effect	4	−0.66 (−1.41, 0.08)	0.081	<0.001	94.5%		0.51
Subgroup analyses of nano-curcumin supplementation on HOMA-IR							
Overall effect	3	−0.28 (−0.33, −0.23)	**<0.001**	0.654	0.0%		0.0
Subgroup analyses of nano-curcumin supplementation on SBP							
Overall effect	5	−7.09 (−12.98, −1.20)	**<0.001**	0.018	83.0%		33.22
Baseline SBP (mmHg)							
<120	1	−2.50 (−11.58, 6.58)	0.590	-	-	0.868	0.0
≥120	4	−8.21 (−15.21, −1.22)	**0.021**	<0.001	87.2%		40.64
Trial duration (week)							
<12	2	−16.65 (−22.98, −10.33)	**<0.001**	0.710	0.0%	<0.001	0.0
≥12	3	−1.19 (−2.39, 0.00)	0.050	0.552	0.0%		0.0
Health status							
Type 2 diabetic	1	−3.60 (−8.27, 1.07)	0.131			<0.001	
Metabolic syndrome	3	−11.98 (−21.29, −2.68)	**0.012**	0.040	68.9%		46.55
Other	1	−1.00 (−2.25, 0.25)	0.118				
BMI baseline							
Overweight (25–29.9 kg/m^2^)	3	−6.79 (−14.20, 0.62)	0.073	0.049	66.8%	0.034	28.45
Obese (>30 kg/m^2^)	2	−8.82 (−25.33, 7.69)	0.295	<0.001	92.3%		131.80
Subgroup analyses of nano-curcumin supplementation on DBP							
Overall effect	3	−0.07 (−1.12, 0.97)	0.891	0.530	0.0%		0.0
Subgroup analyses of nano-curcumin supplementation on CRP							
Overall effect	7	−1.29 (−2.15, −0.44)	**0.003**	<0.001	87.0%		1.02
Baseline CRP (mg/L)							
<3	2	−0.64 (−1.52, 0.23)	0.152	0.007	86.2%	0.001	0.34
≥3	5	−1.71 (−3.08, −0.35)	**0.014**	<0.001	85.2%		1.98
Trial duration (week)							
<12	4	−0.57 (−1.17, 0.01)	0.057	0.054	60.8%	<0.001	0.19
≥12	3	−2.61 (−4.58, −0.64)	**0.009**	<0.001	89.2%		2.58
Health status							
Type 2 diabetic	1	−0.70 (−1.72, 0.32)	0.181	-	-	0.000	0.0
Metabolic syndrome	2	−0.64 (−1.52, −1.52)	0.152	0.007	86.2%		0.34
Migraine	2	−0.39 (−1.35, 0.55)	0.415	0.611	0.0%		0.0
Other	2	−3.78 (−7.15, −0.41)	**0.028**	0.002	89.7%		5.33
BMI baseline							
Overweight (25–29.9 kg/m^2^)	5	−1.40 (−2.55, −0.25)	**0.017**	<0.001	81.5%	0.286	1.30
Obese (>30 kg/m^2^)	2	−1.18 (−3.14, 0.77)	0.237	<0.001	95.7%		1.91
Subgroup analyses of nano-curcumin supplementation on IL-6							
Overall effect	5	−2.78 (−3.76, −1.79)	**<0.001**	0.627	0.0%		0.0
Subgroup analyses of nano-curcumin supplementation on TNF-α							
Overall effect	2	−3.09 (−8.75, 2.57)	0.284	<0.001	99.1%		16.55
Subgroup analyses of nano-curcumin supplementation on body mass							
Overall effect	5	−0.51 (−1.85, 0.82)	0.449	0.974	0.0%		0.0
Subgroup analyses of nano-curcumin supplementation on BMI							
Overall effect	7	−0.35 (−0.76, 0.04)	0.079	0.207	29.0%		0.13
Subgroup analyses of nano-curcumin supplementation on WC							
Overall effect	5	−1.32 (−3.89, 1.23)	0.310	0.015	67.4%		5.26
Subgroup analyses of nano-curcumin supplementation on FM							
Overall effect	4	−0.86 (−1.95, 0.23)	0.123	0.371	4.3%		0.06

Abbreviations: CI, confidence interval; WMD, weighted mean differences; TG. Triglycerides; TC, total cholestrol; LDL-C, low-density lipoprotein; HDL-C, high-density lipoprotein; FBG, fasting blood glucose; HbA1c, hemoglobin A1c; HOMA-IR, homeostatic model assessment for insulin resistance; SBP, systolic blood pressure; DBP, diastolic blood pressure; CRP, C-reactive protein, IL-6, interlukin 6; TNF-α; tumor necrosis factor α; BMI, body mass index; WC, waist circumference; FM, fat mass.

**Table 3:** GRADE profile of nano-curcumin supplementation for cardiovasscular risk factors scores in adults.

Quality Assessment						Summary of Findings			Quality of Evidence
Outcomes	Risk of Bias	Inconsistency	Indirectness	Imprecision	Publication Bias	Number of Intervention/Control	WMD (95%CI)	Heterogeneity (I^2^)	
TG	No serious limitations	Very serious Limitations	No serious limitations	Serious Limitations	No serious limitations	174/163	−9.76 (−32.71, 13.17)	79.2%	⊕◯◯◯ Very low
TC	No serious limitations	Very serious Limitations	No serious limitations	Serious Limitations	No serious limitations	152/152	−3.34 (−14.43, 7.73)	77.2%	⊕◯◯◯ Very low
LDL-C	No serious limitations	Very serious Limitations	No serious limitations	Serious Limitations	No serious limitations	152/152	−3.59 (−15.74, 8.56)	84.8%	⊕◯◯◯ Very low
HDL-C	No serious limitations	Very serious Limitations	No serious limitations	No serious limitations	No serious limitations	174/163	5.77 (2.90, 8.64)	83.5%	⊕⊕◯◯ Low
FBG	No serious limitations	Very serious Limitations	No serious limitations	No serious limitations	Serious Limitations	214/203	−18.14 (−29.31, −6.97)	84.9%	⊕◯◯◯ Very low
Fasting insulin	No serious limitations	No serious limitations	No serious limitations	No serious limitations	No serious limitations	90/90	−1.21 (−1.43, −1.00)	0.0%	⊕⊕⊕⊕ High
HbA1c	No serious limitations	Very serious Limitations	No serious limitations	Serious Limitations	No serious limitations	139/138	−0.66 (−1.41, 0.08)	94.5%	⊕◯◯◯ Very low
HOMA-IR	No serious limitations	No serious limitations	No serious limitations	No serious limitations	No serious limitations	90/90	−0.28 (−0.33, −0.23)	0.0%	⊕⊕⊕⊕ High
SBP	No serious limitations	Very serious Limitations	No serious limitations	No serious limitations	No serious limitations	112/101	−7.09 (−12.98, −1.20)	83.2%	⊕⊕◯◯ Low
DBP	No serious limitations	No serious limitations	No serious limitations	Serious Limitations	No serious limitations	90/90	−0.07 (−1.12, 0.97)	0.0%	⊕⊕⊕◯ Moderate
CRP	No serious limitations	Very serious Limitations	No serious limitations	No serious limitations	No serious limitations	153/126	−1.29 (−2.15, −0.44)	87.0%	⊕⊕◯◯ Low
IL-6	No serious limitations	No serious limitations	No serious limitations	No serious limitations	No serious limitations	100/72	−2.78 (−3.76, −1.79)	0.0%	⊕⊕⊕⊕ High
TNF-α	No serious limitations	Very serious Limitations	No serious limitations	Serious Limitations	No serious limitations	61/61	−3.09 (−8.75, 2.57)	99.1%	⊕◯◯◯ Very low
Body weight	No serious limitations	No serious limitations	No serious limitations	Serious Limitations	Serious Limitations	126/114	−0.51 (−1.85, 0.82)	0.0%	⊕⊕◯◯ Low
BMI	No serious limitations	No serious limitations	No serious limitations	Serious Limitations	No serious limitations	188/176	−0.35 (−0.76, 0.04)	29.0%	⊕⊕⊕◯ Moderate
WC	No serious limitations	Serious Limitations	No serious limitations	Serious Limitations	No serious limitations	126/114	−1.32 (−3.89, 1.23)	67.4%	⊕⊕◯◯ Low
FM	No serious limitations	No serious limitations	No serious limitations	Serious Limitations	No serious limitations	86/74	−0.86 (−1.95, 0.23)	4.3%	⊕⊕⊕◯ Moderate

Abbreviations: TG. Triglycerides; TC, total cholesterol; LDL-C, low-density lipoprotein; HDL-C, high-density lipoprotein; FBG, fasting blood glucose; HbA1c, hemoglobin A1c; HOMA-IR, homeostatic model assessment for insulin resistance; SBP, systolic blood pressure; DBP, diastolic blood pressure; CRP, C-reactive protein, IL-6, interleukin 6; TNF-α; tumor necrosis factor α; BMI, body mass index; WC, waist circumference; FM, fat mass.

## Supplementary Materials

The following are available online at https://www.mdpi.com/article/10.3390/antiox10071015/s1.
