# Supplementary material for: The Effects of Nano-Curcumin Supplementation on Risk Factors for Cardiovascular Disease: A GRADE-Assessed Systematic Review and Meta-Analysis of Clinical Trials
Source: Antioxidants (Basel). 2021 Jun 24;10(7):1015. doi: 10.3390/antiox10071015 (PMC8300831; doi:10.3390/antiox10071015)
Supplement: Supplementary file 1 [file antioxidants-10-01015-s001.zip › antioxidants-1255465-supplementary.pdf]

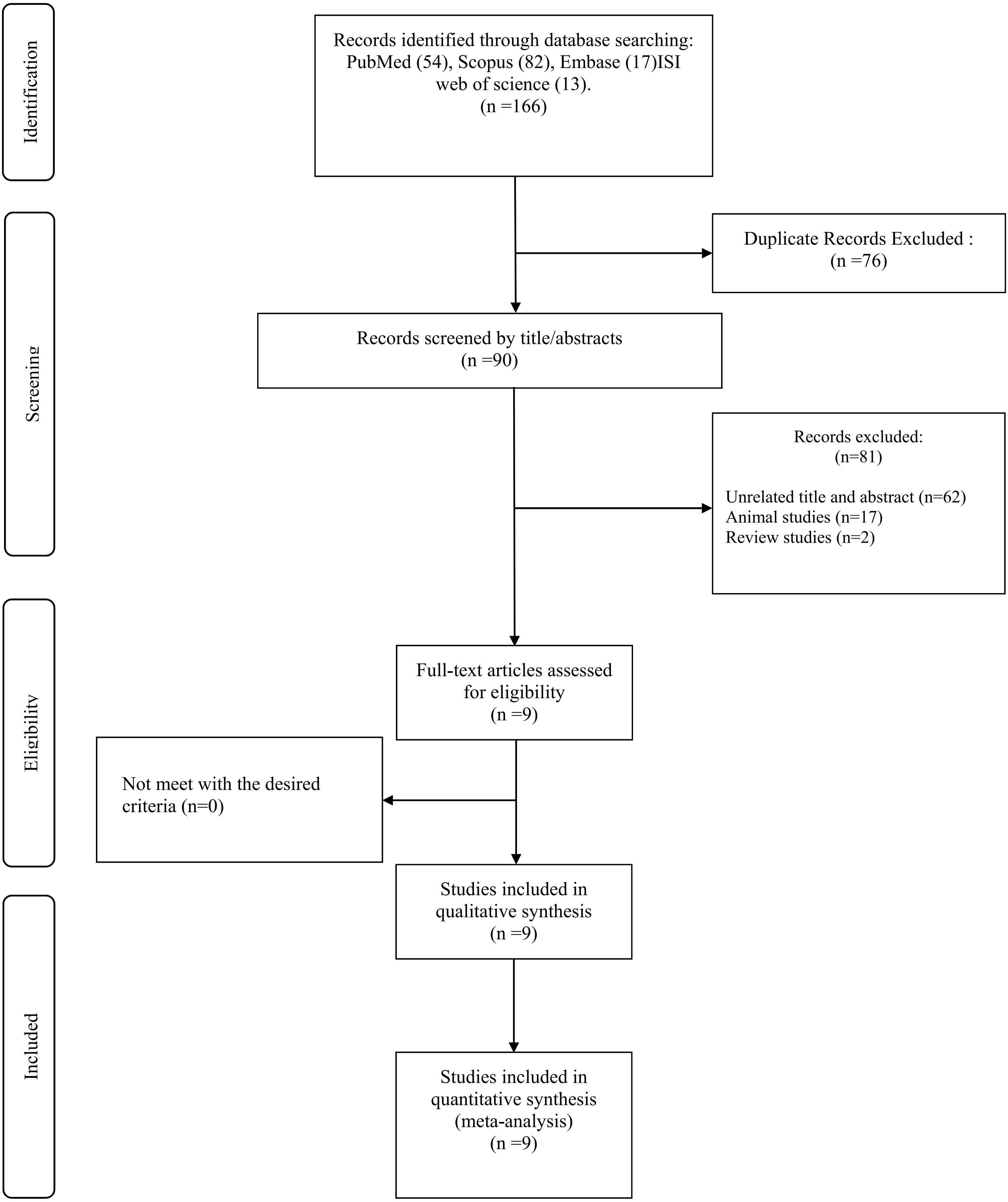

Supplementary Figure S1. Flowchart of study selection for inclusion trials in the systematic review.

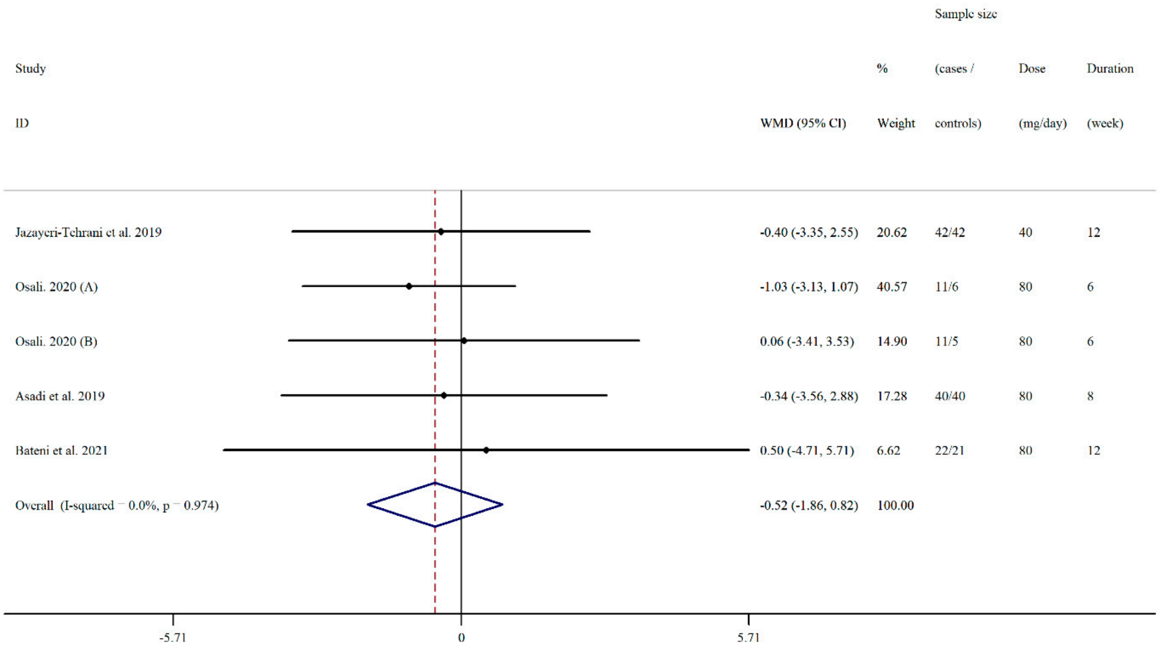

Supplementary Figure S2. Forest plot of the random-effects meta-analysis of the effect of nano-curcumin on body weight.

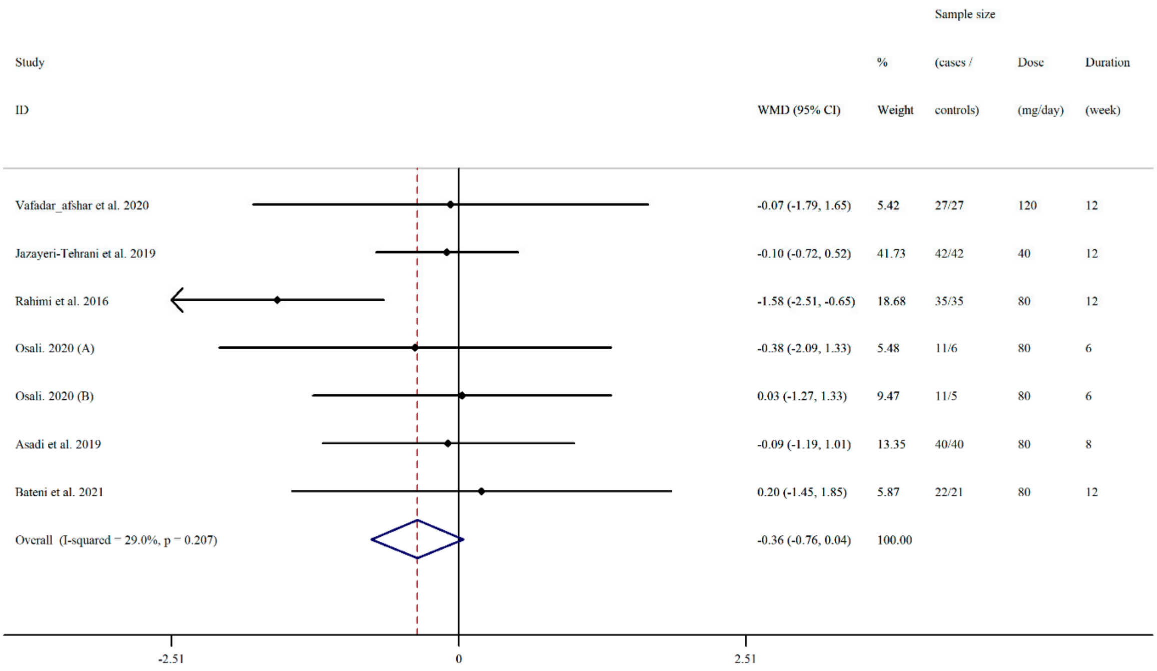

Supplementary Figure S3. Forest plot of the random-effects meta-analysis of the effect of nano-curcumin on BMI.

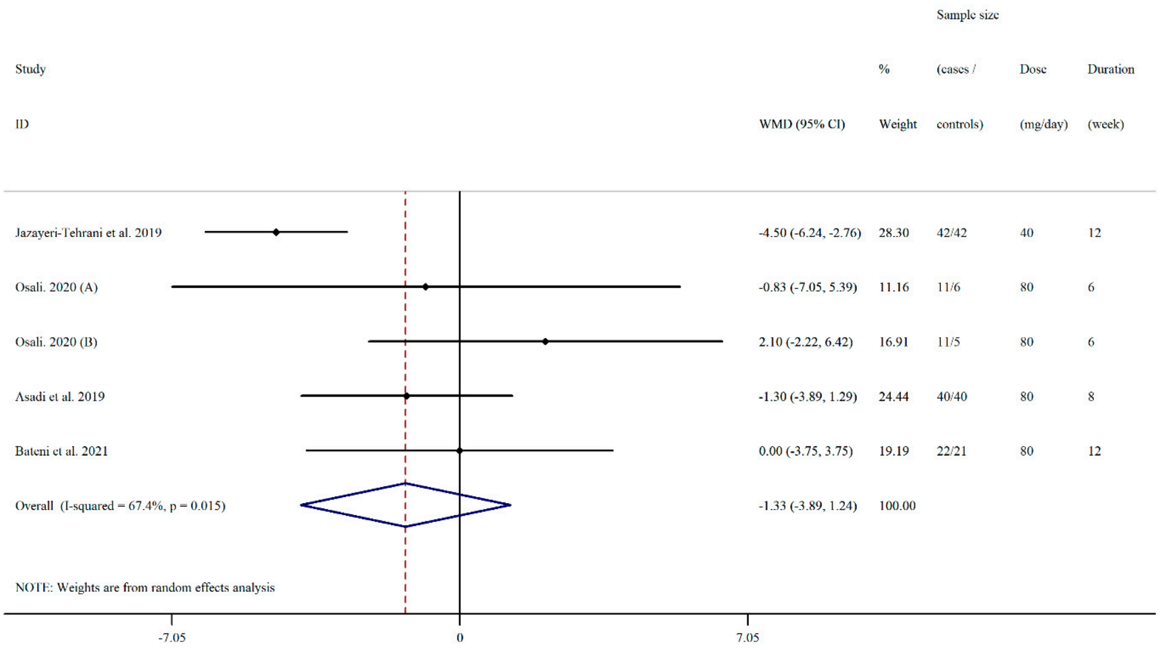

Supplementary Figure S4. Forest plot of the random-effects meta-analysis of the effect of nano-curcumin on WC.

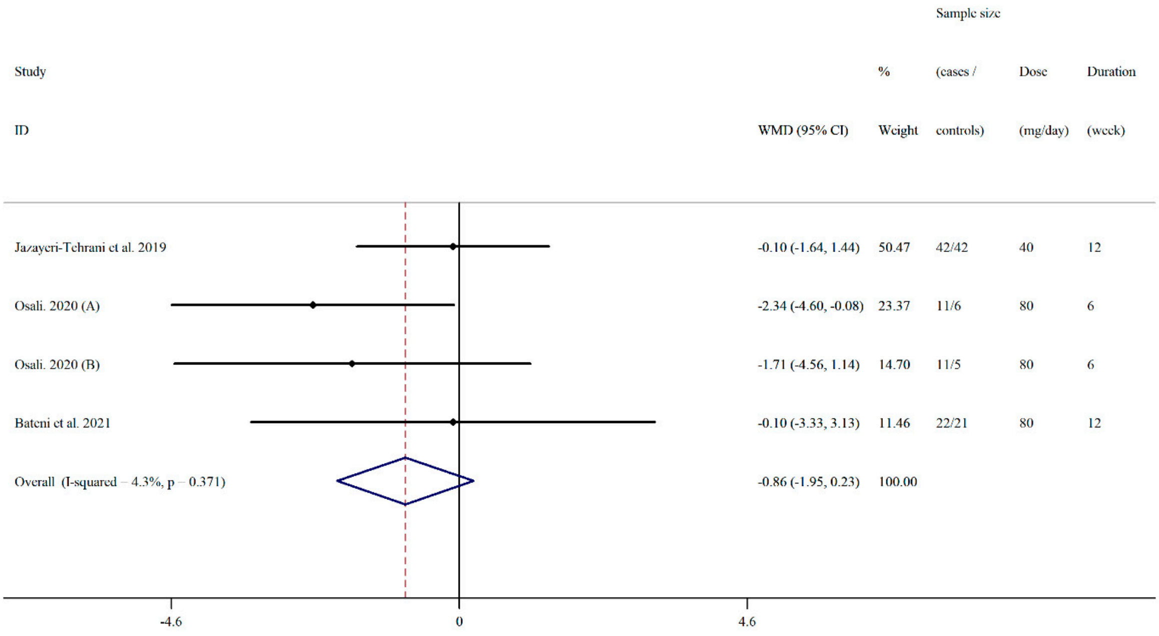

Supplementary Figure S5. Forest plot of the random-effects meta-analysis of the effect of nano-curcumin on FM.

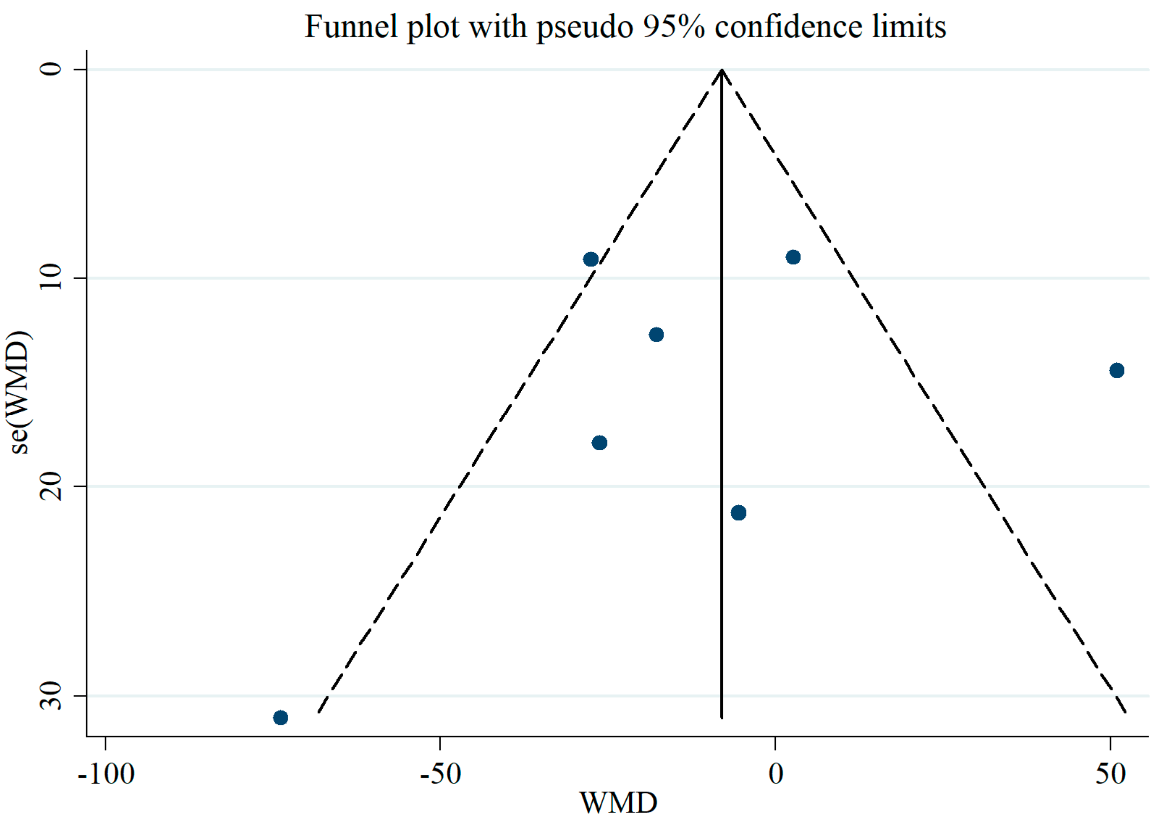

Supplementary Figure S6. Funnel plot representing publication bias in the studies reporting the effect of nano-curcumin on TG.

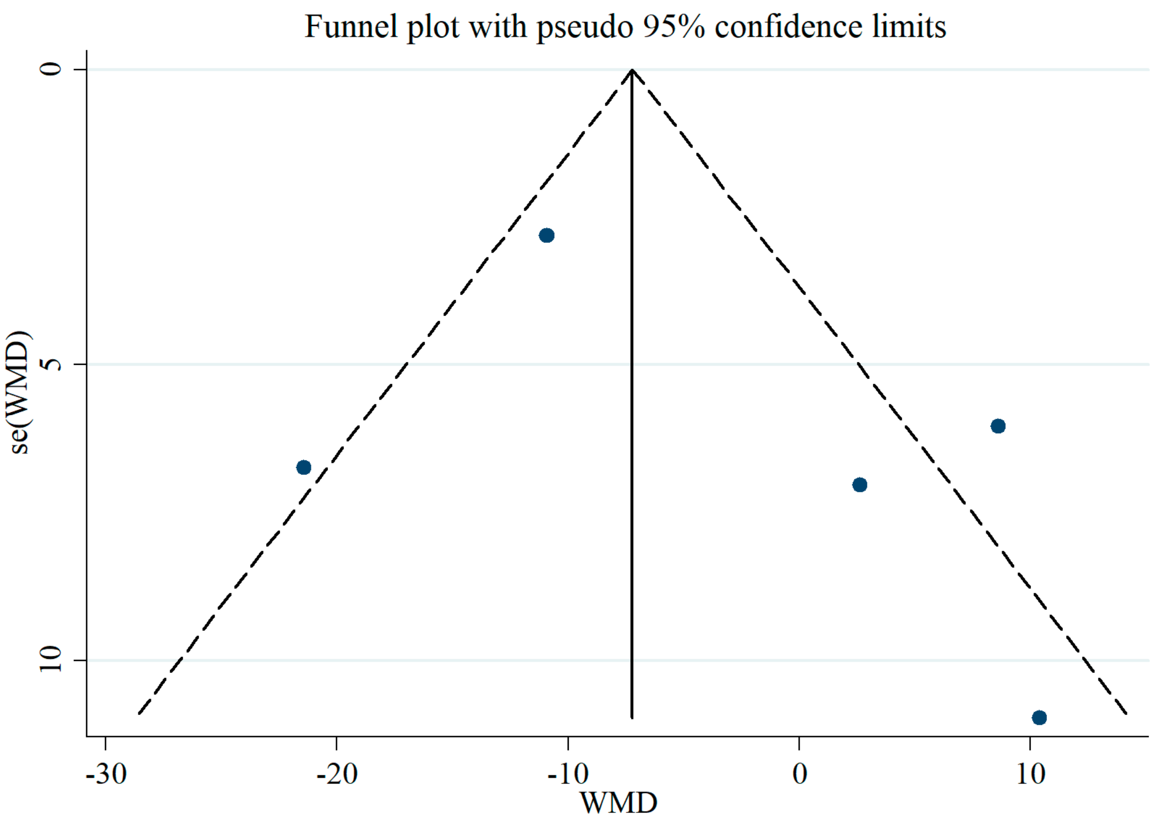

Supplementary Figure S7. Funnel plot representing publication bias in the studies reporting the effect of nano-curcumin on TC.

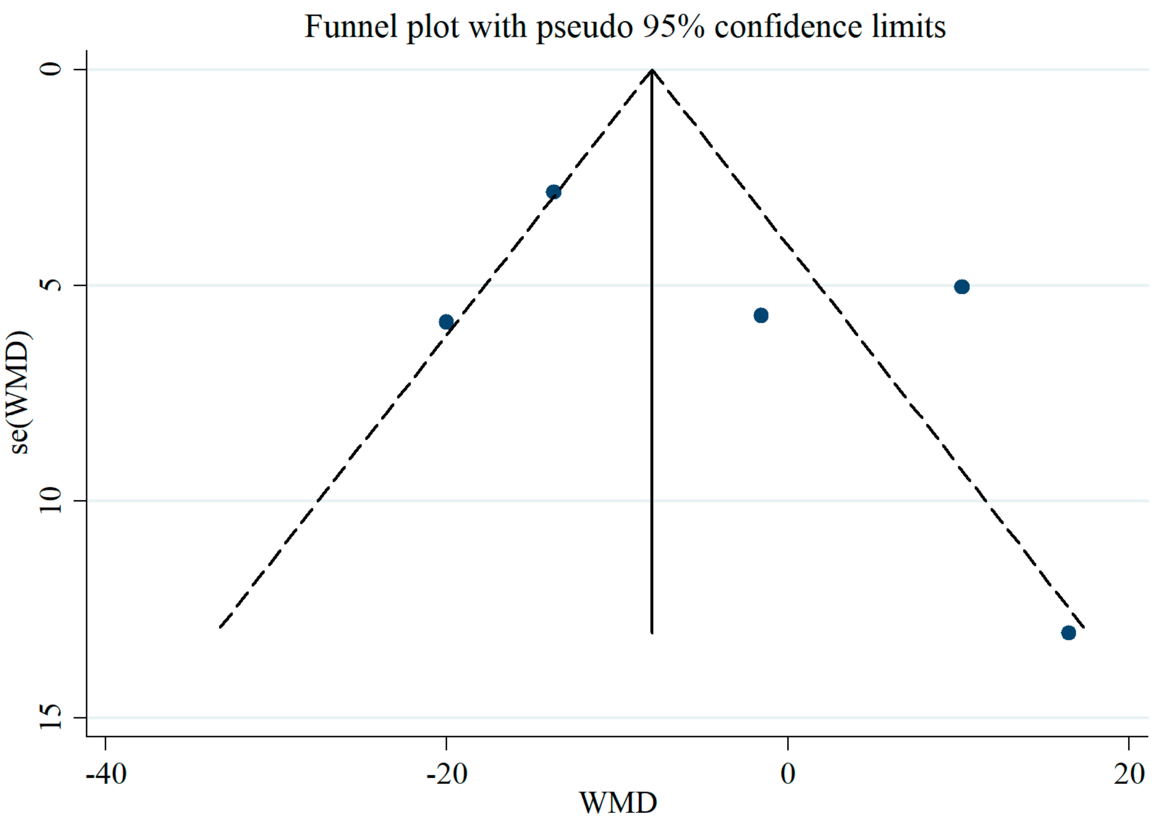

Supplementary Figure S8. Funnel plot representing publication bias in the studies reporting the effect of nano-curcumin on LDL.

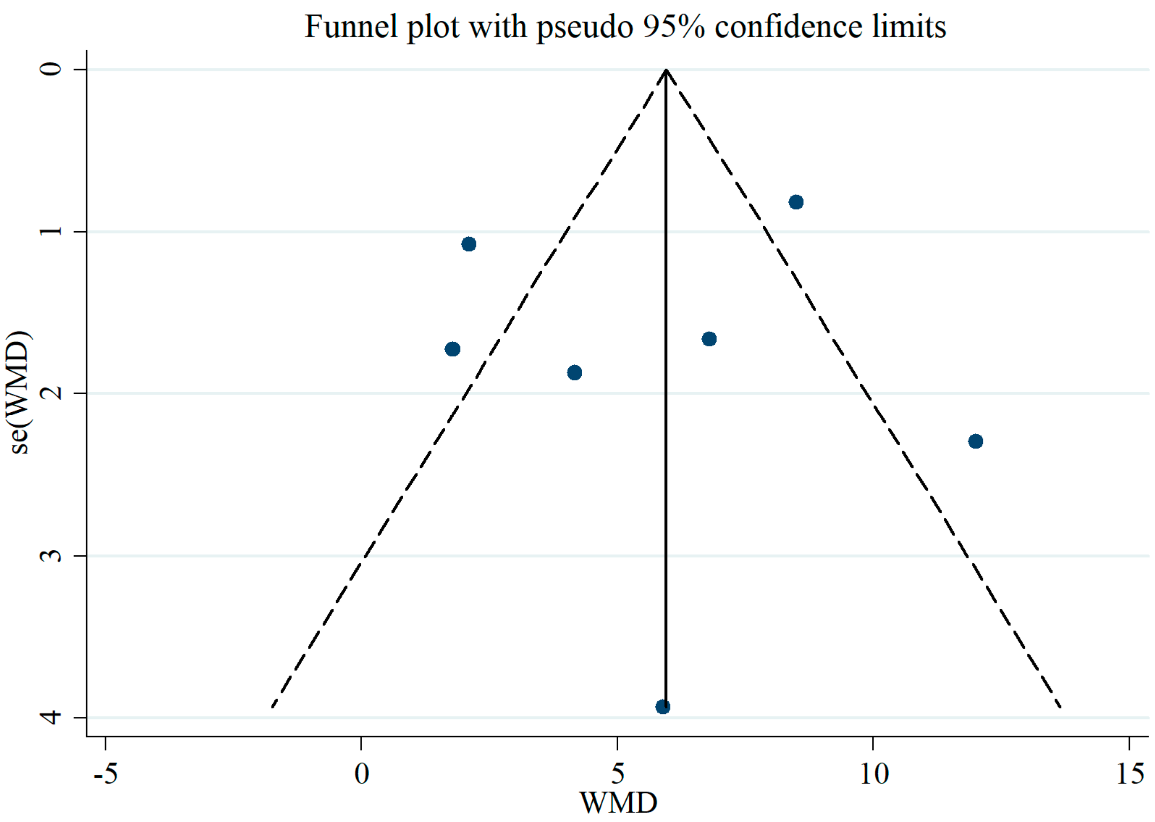

Supplementary Figure S9. Funnel plot representing publication bias in the studies reporting the effect of nano-curcumin on HDL.

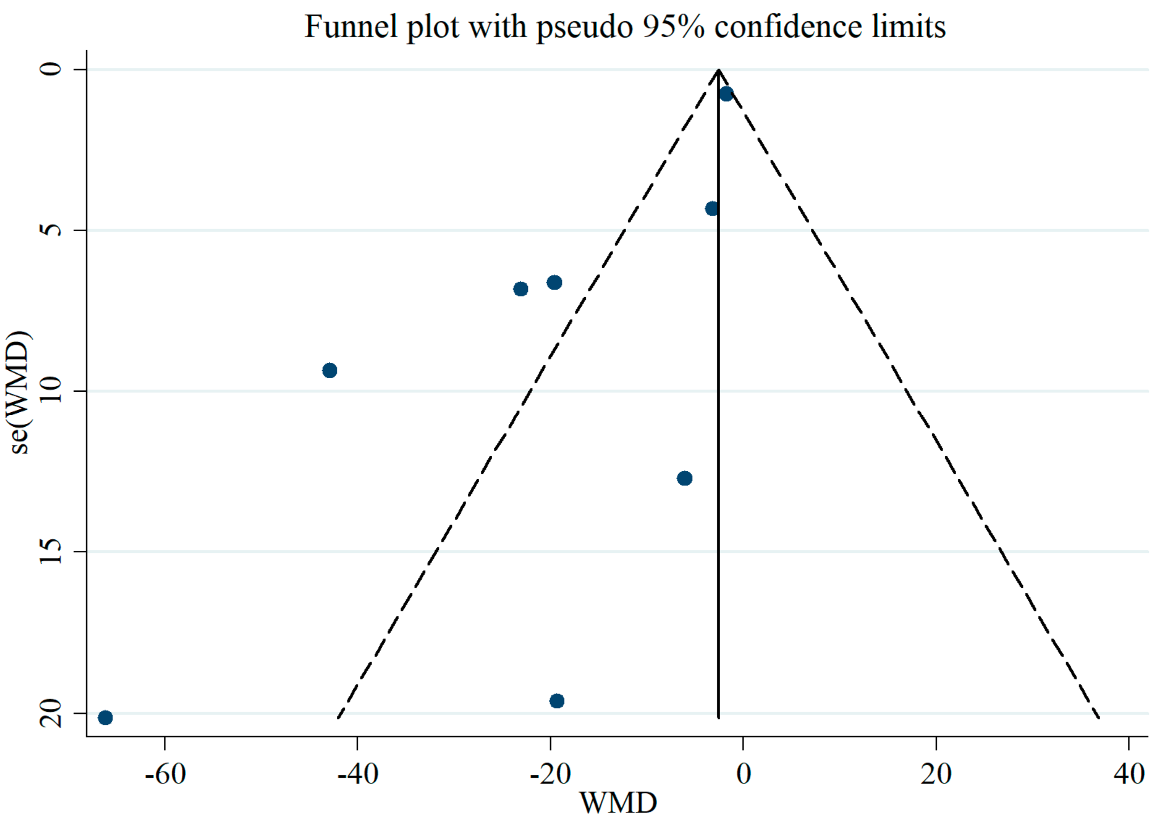

Supplementary Figure S10. Funnel plot representing publication bias in the studies reporting the effect of nano-curcumin on FBS.

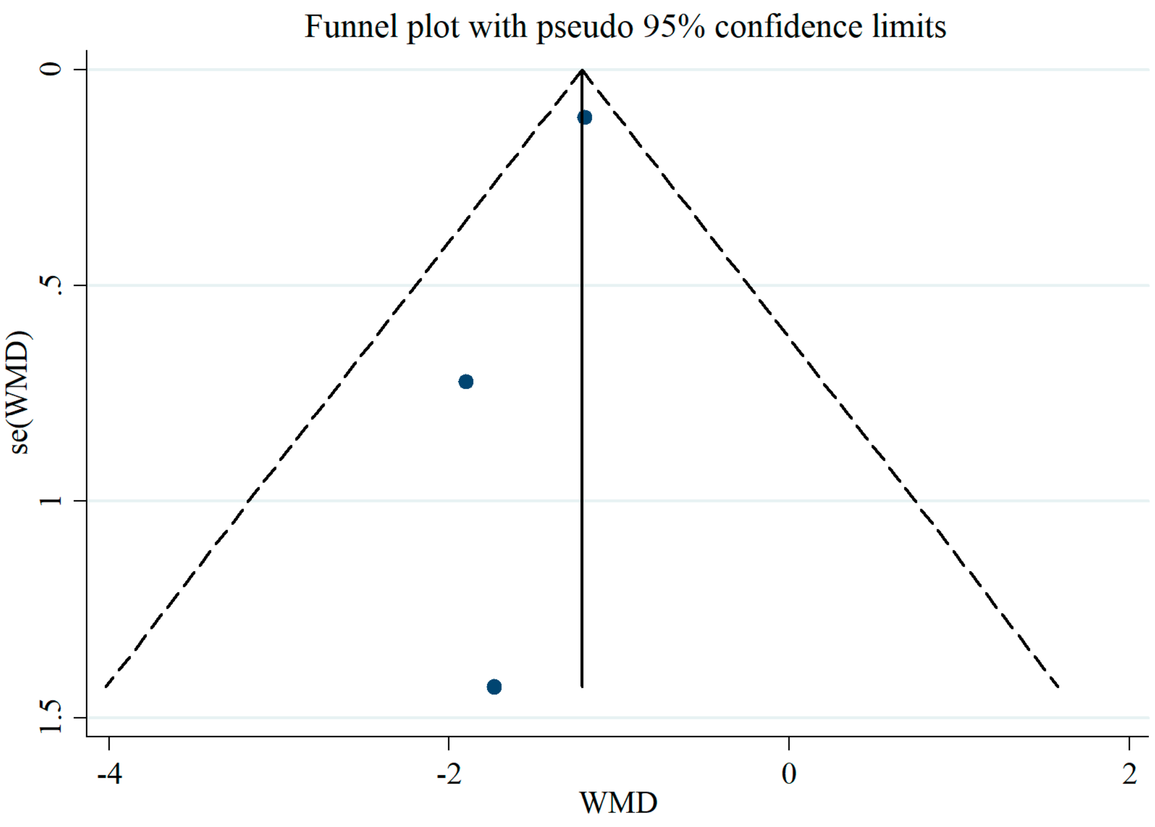

Supplementary Figure S11. Funnel plot representing publication bias in the studies reporting the effect of nano-curcumin on fasting insulin.

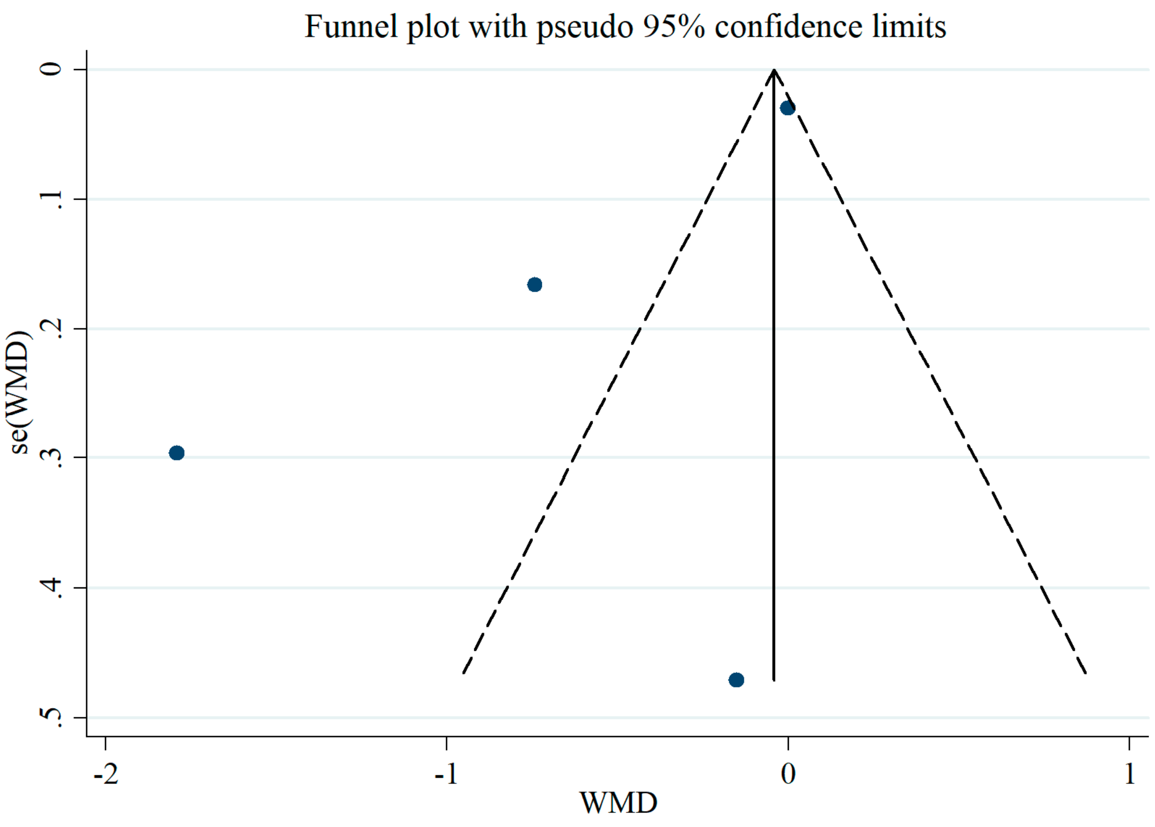

Supplementary Figure S12. Funnel plot representing publication bias in the studies reporting the effect of nano-curcumin on HbA1c.

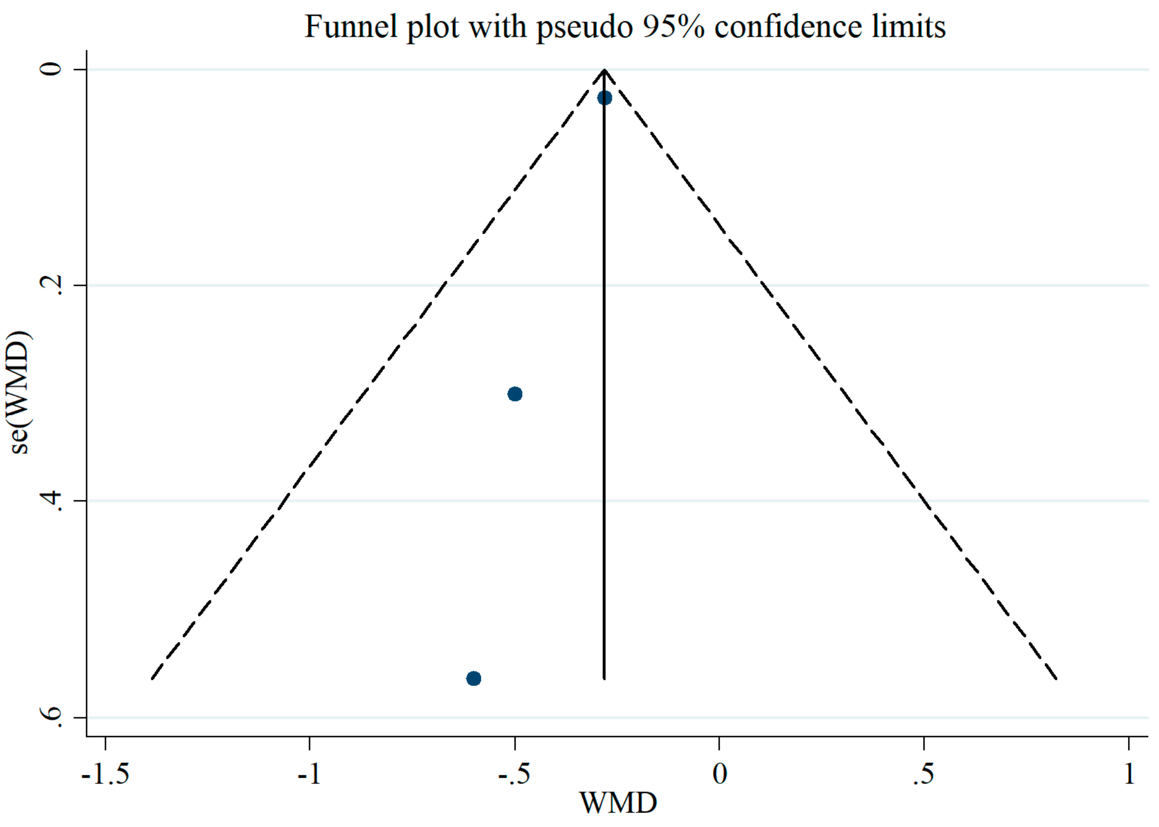

Supplementary Figure S13. Funnel plot representing publication bias in the studies reporting the effect of nano-curcumin on HOMA-IR.

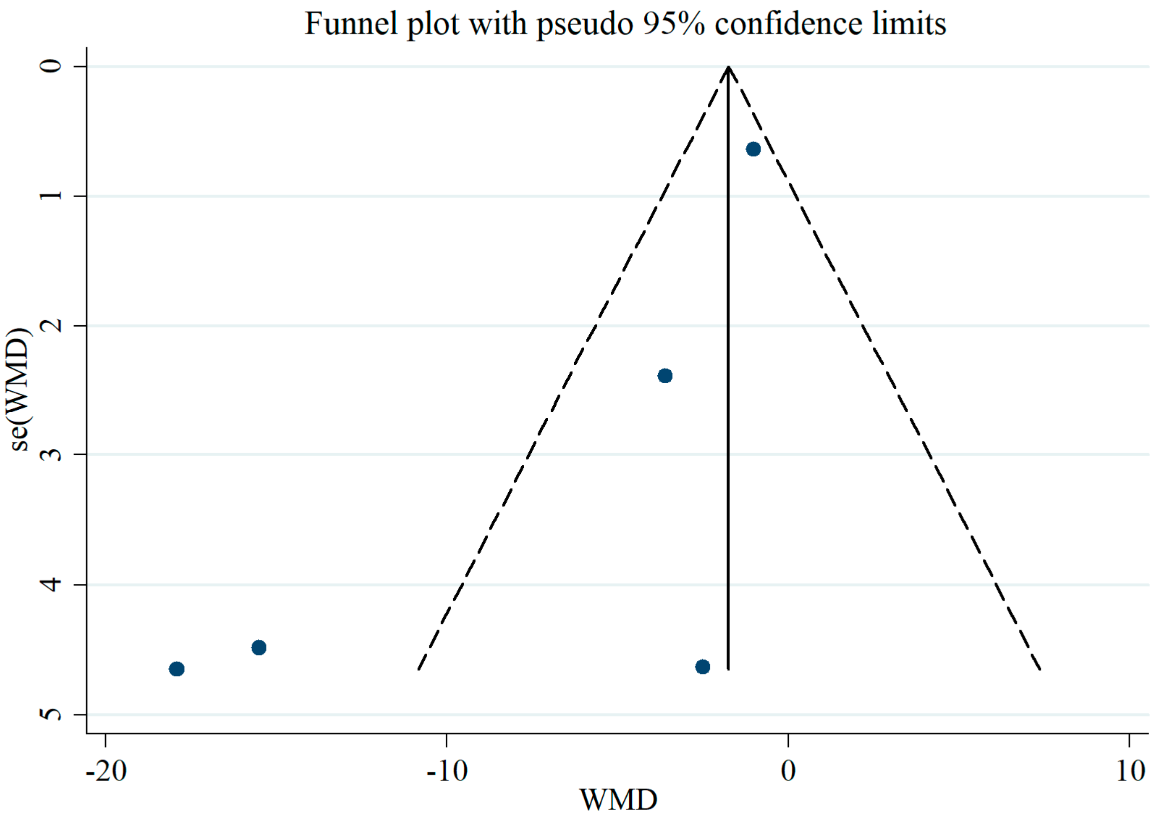

Supplementary Figure S14. Funnel plot representing publication bias in the studies reporting the effect of nano-curcumin on SBP.

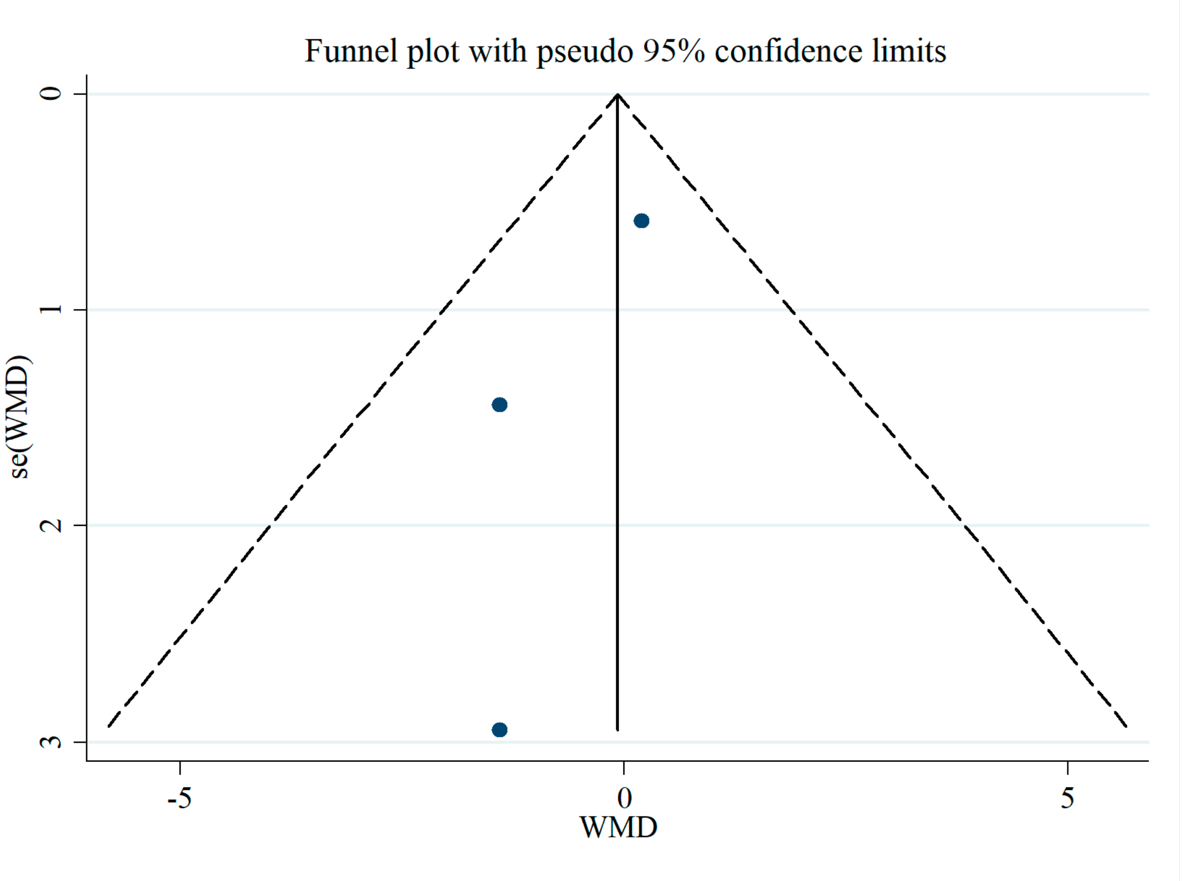

Supplementary Figure S15. Funnel plot representing publication bias in the studies reporting the effect of nano-curcumin on DBP.

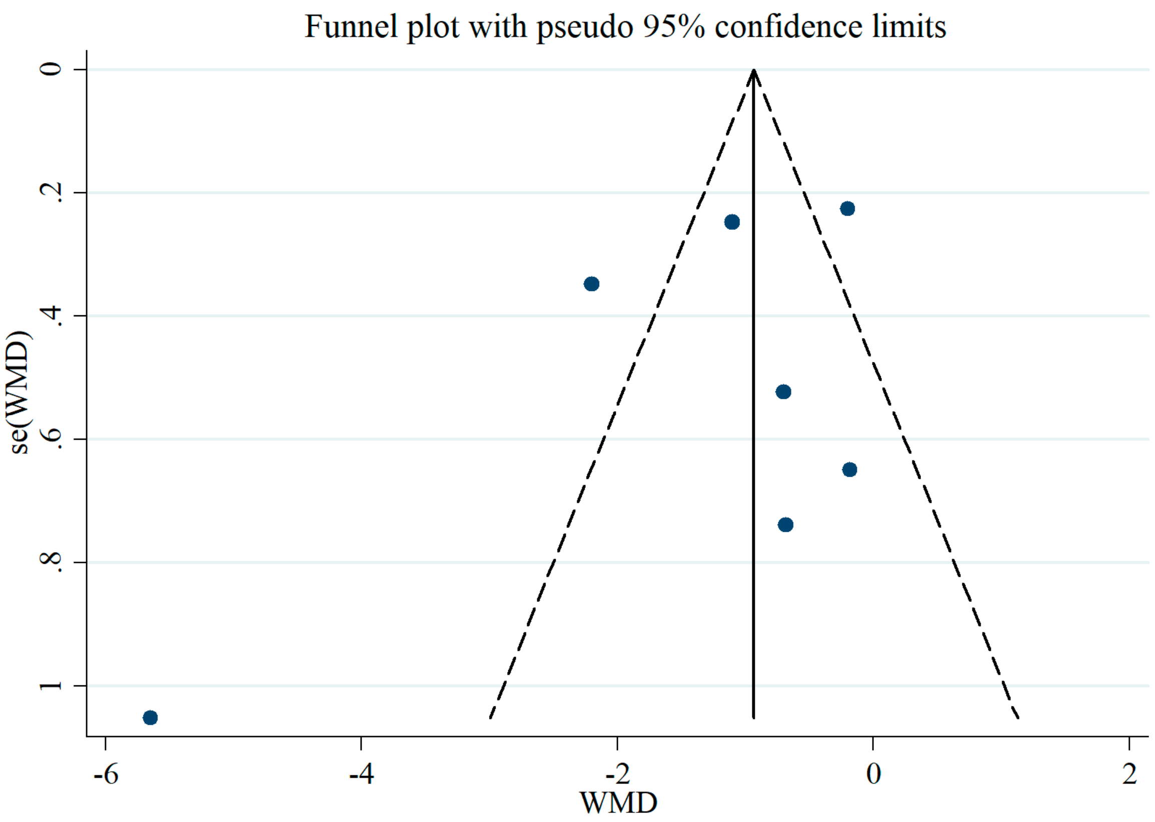

Supplementary Figure S16. Funnel plot representing publication bias in the studies reporting the effect of nano-curcumin on CRP.

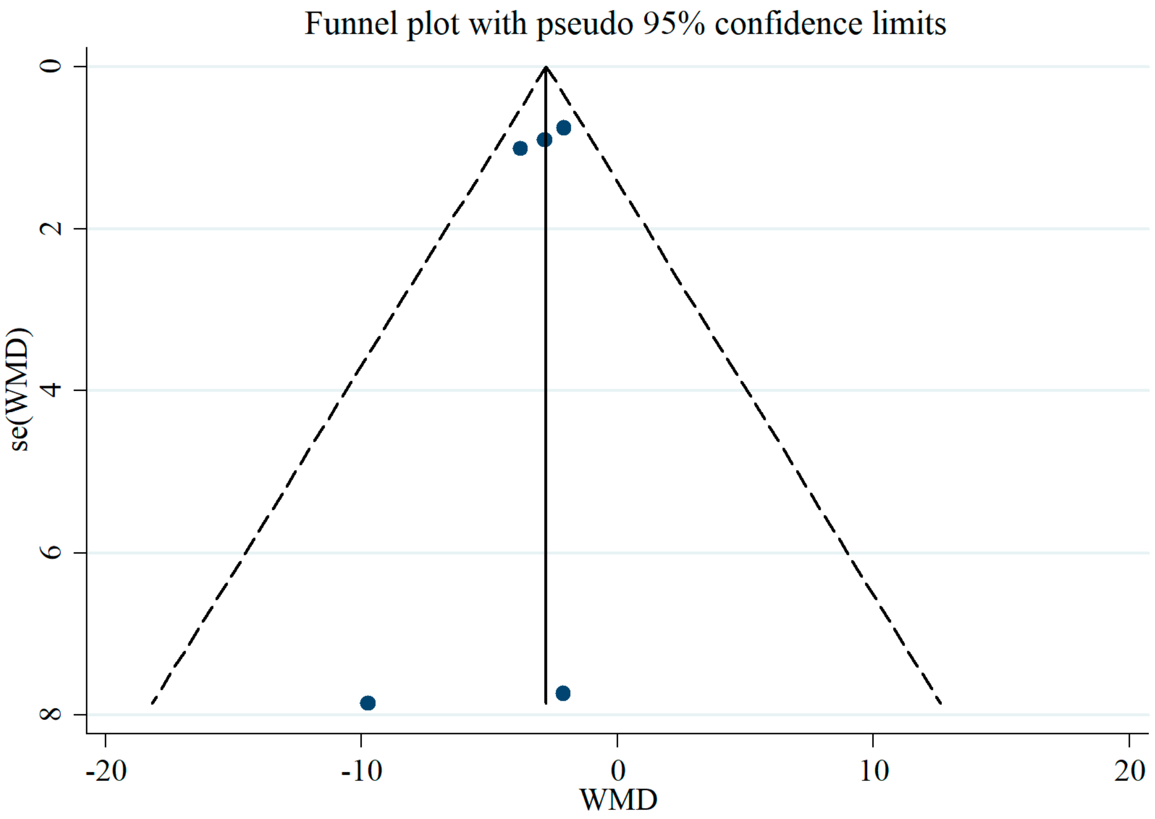

Supplementary Figure S17. Funnel plot representing publication bias in the studies reporting the effect of nano-curcumin on IL-6.

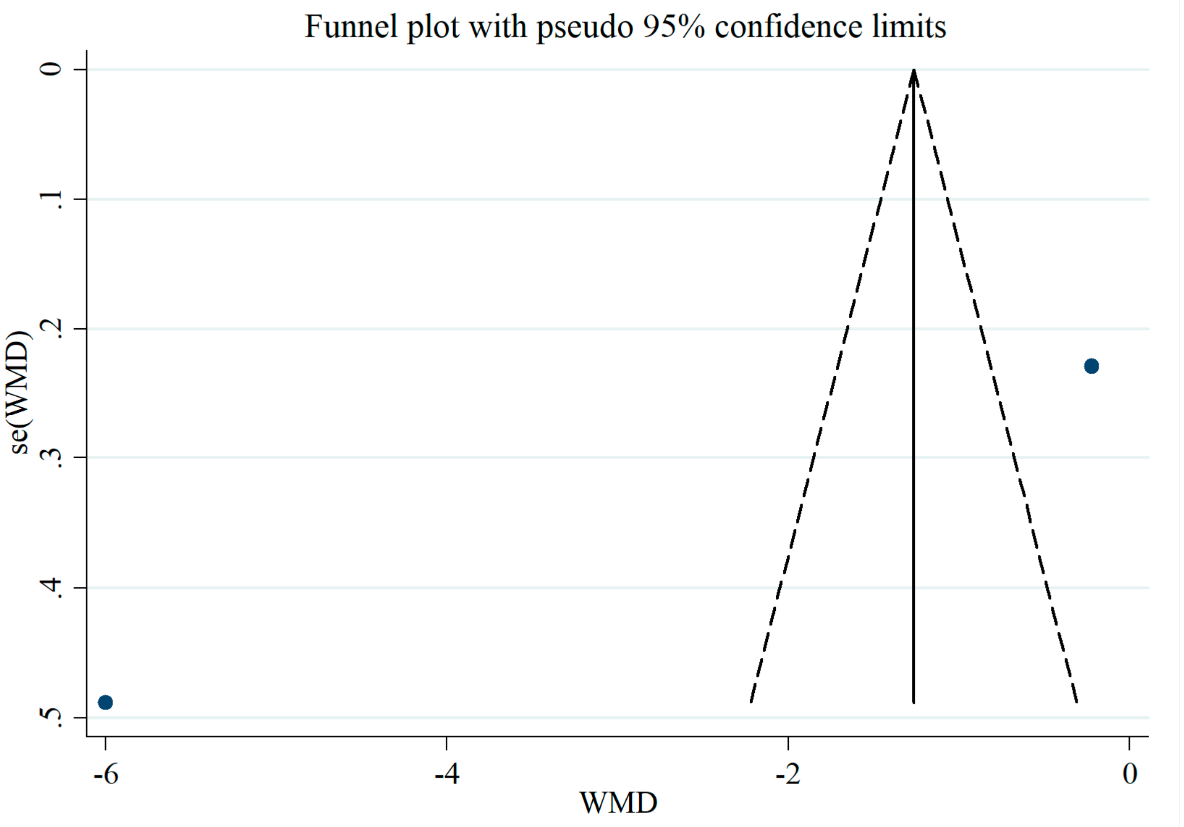

Supplementary Figure S18. Funnel plot representing publication bias in the studies reporting the effect of nano-curcumin on TNF-α.

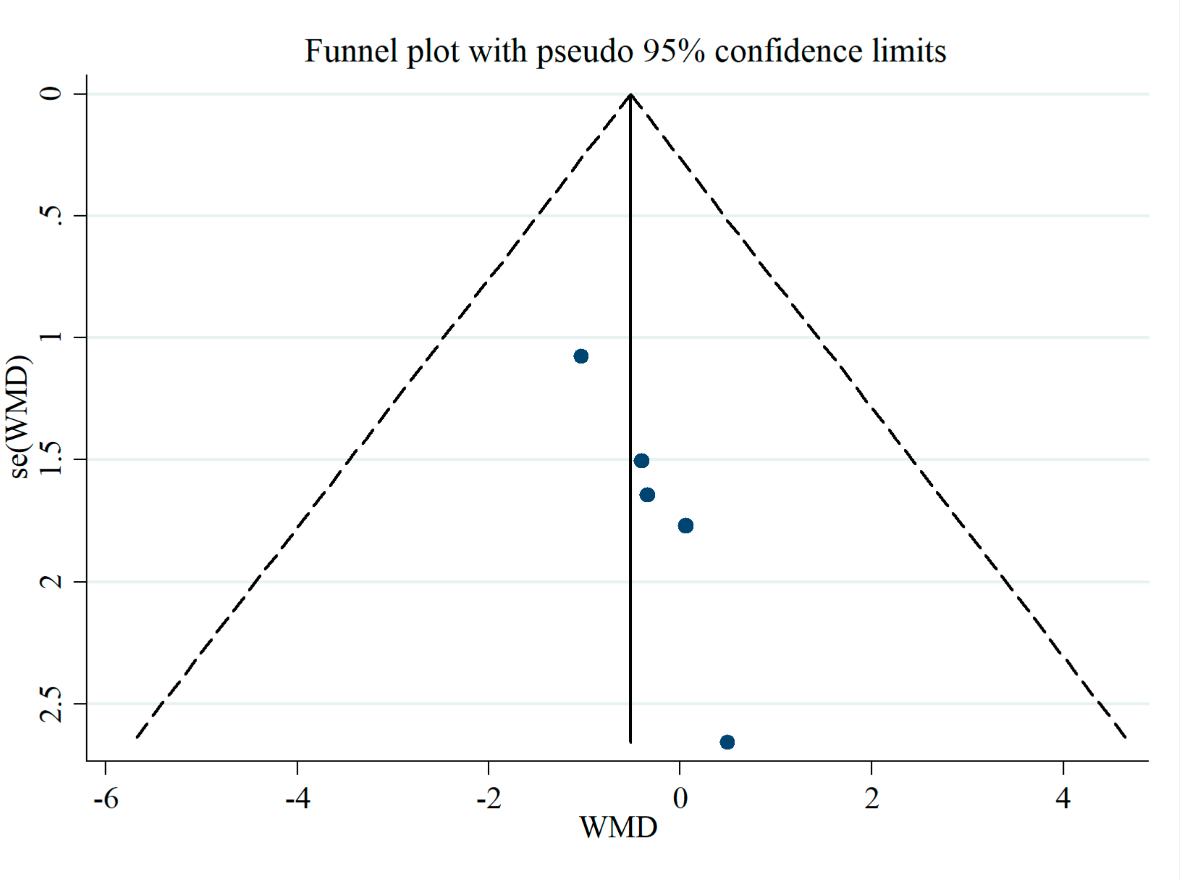

Supplementary Figure S19. Funnel plot representing publication bias in the studies reporting the effect of nano-curcumin on body weight.

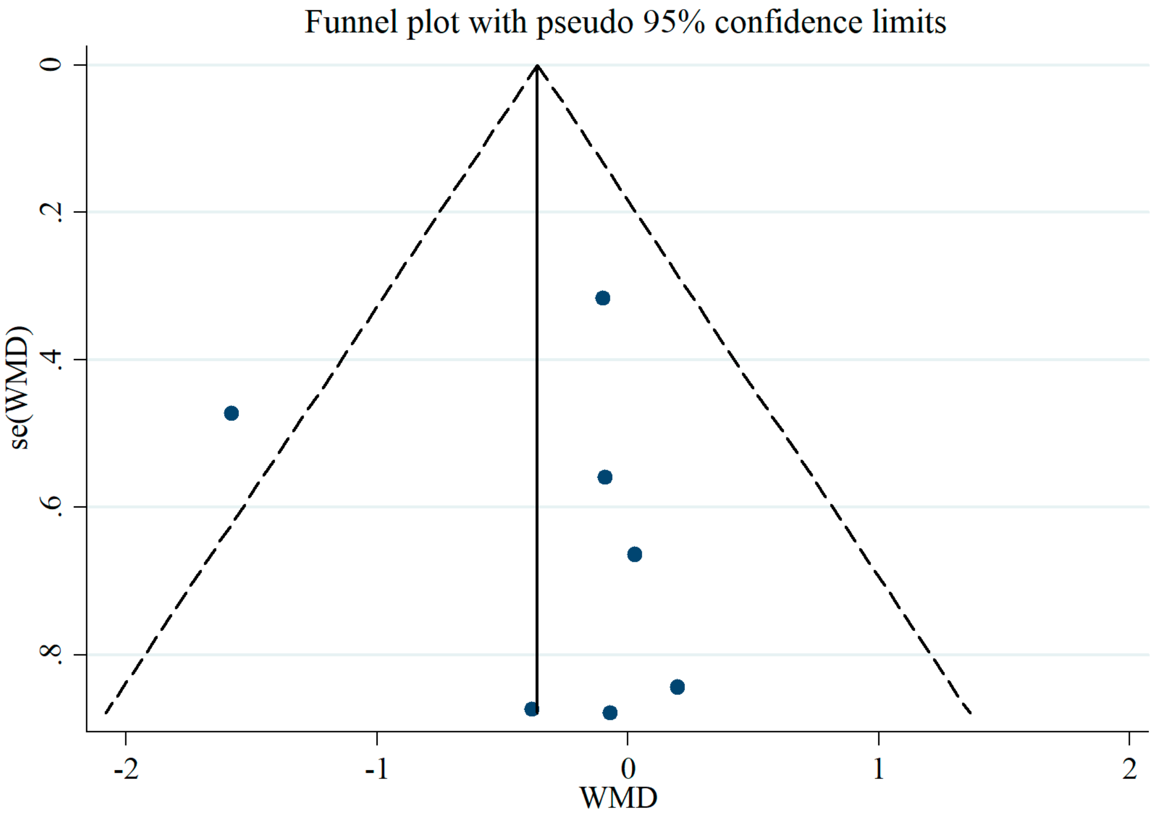

Supplementary Figure S20. Funnel plot representing publication bias in the studies reporting the effect of nano-curcumin on BMI.

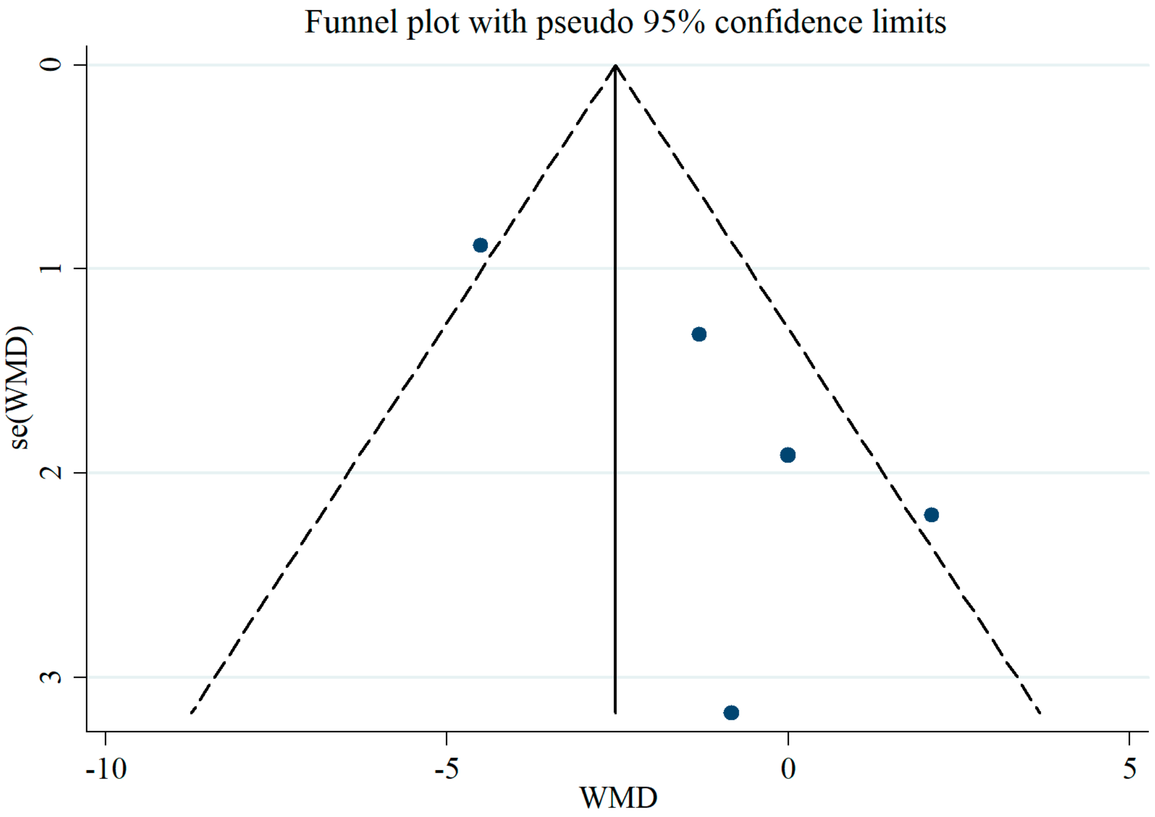

Supplementary Figure S21. Funnel plot representing publication bias in the studies reporting the effect of nano-curcumin on WC.

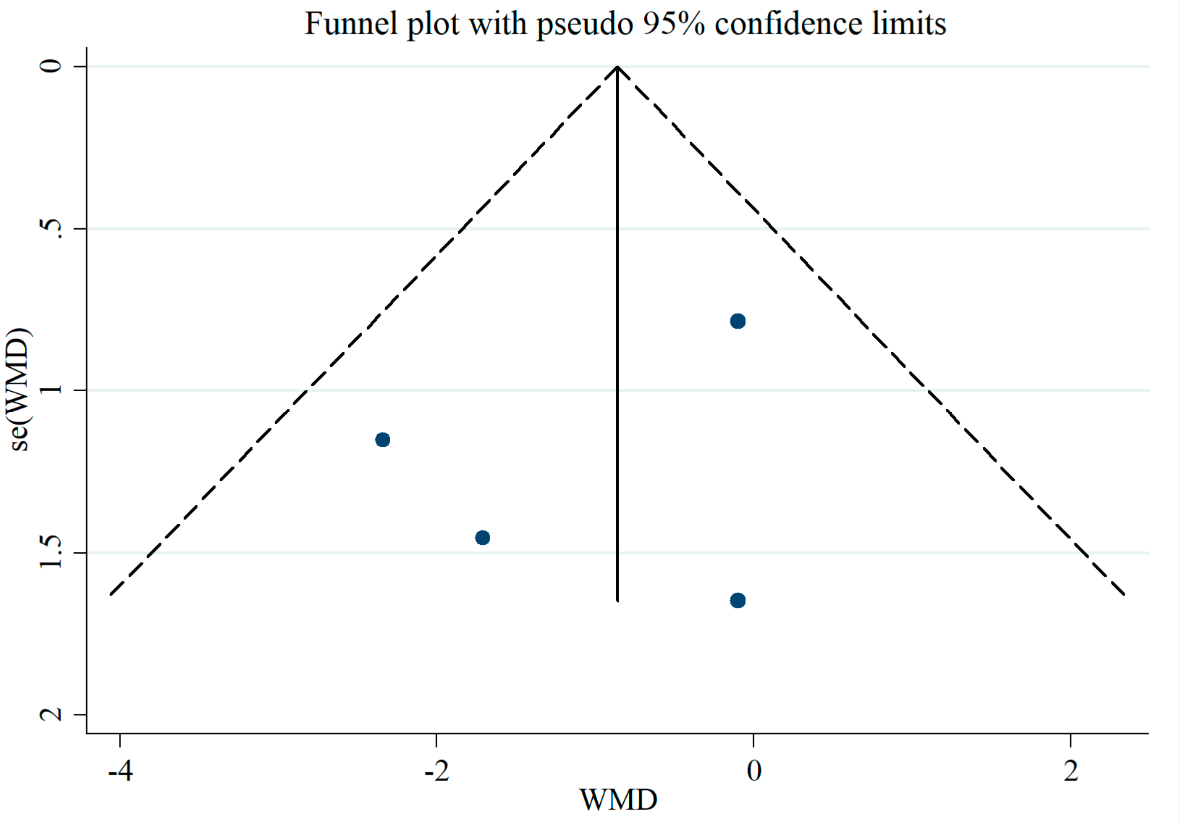

Supplementary Figure S22. Funnel plot representing publication bias in the studies reporting the effect of nano-curcumin on FM.

Supplementary Table S1. Quality assessment of included studies.

studies	Random sequence generation	Allocation concealment	Selective reporting	Other sources of bias	Blinding (participants and personnel)	Blinding (outcome assessment)	Incomplete outcome data
Vafadar_afshar et al. 2020	L	U	H	U	L	U	L
Jazayeri-Tehrani et al. 2019	L	U	H	U	L	L	L
Abdolahi et al. 2017	L	L	H	U	L	U	L
Rahimi et al. 2016	L	U	H	U	L	L	L
Osali. 2020	L	L	H	U	L	L	L
Asadi et al. 2019	L	U	H	U	L	U	L
Bateni et al. 2021	L	U	H	U	L	U	L
Abdolahi et al. 2018	L	L	H	U	L	U	L
Shafabakhsh et al. 2020	L	L	H	U	L	L	L

Abbreviations: L, low risk of bias; H, high risk of bias; U, unclear risk of bias.
